# Genomic context– and H2AK119 ubiquitination–dependent inheritance of human Polycomb silencing

**DOI:** 10.1126/sciadv.adl4529

**Published:** 2024-05-08

**Authors:** Tiasha A. Shafiq, Juntao Yu, Wenzhi Feng, Yizhe Zhang, Haining Zhou, Joao A. Paulo, Steven P. Gygi, Danesh Moazed

**Affiliations:** ^1^ Department of Cell Biology, Howard Hughes Medical Institute, Blavatnik Institute, Harvard Medical School, Boston, MA, USA.; ^2^ Department of Cell Biology, Blavatnik Institute, Harvard Medical School, Boston, MA, USA.

## Abstract

Polycomb repressive complexes 1 and 2 (PRC1 and 2) are required for heritable repression of developmental genes. The cis- and trans-acting factors that contribute to epigenetic inheritance of mammalian Polycomb repression are not fully understood. Here, we show that, in human cells, ectopically induced Polycomb silencing at initially active developmental genes, but not near ubiquitously expressed housekeeping genes, is inherited for many cell divisions. Unexpectedly, silencing is heritable in cells with mutations in the H3K27me3 binding pocket of the Embryonic Ectoderm Development (EED) subunit of PRC2, which are known to disrupt H3K27me3 recognition and lead to loss of H3K27me3. This mode of inheritance is less stable and requires intact PRC2 and recognition of H2AK119ub1 by PRC1. Our findings suggest that maintenance of Polycomb silencing is sensitive to local genomic context and can be mediated by PRC1-dependent H2AK119ub1 and PRC2 independently of H3K27me3 recognition.

## Teaser

Heritable Polycomb silencing is sensitive to genomic context and relies on positive feedback from distinct histone modifications.

## INTRODUCTION

Multicellular organisms are composed of many cell types, nearly all of which have the same genome, but express different gene expression programs. These programs are directed by lineage- and cell type–specific transcription factor networks (TFNs), which form potent autoregulatory loops that must be silenced in other cell types (*1*–*4*). The Polycomb group (PcG) proteins form histone-modifying repressor complexes that silence cell type–specific transcription factors outside their proper spatial domains of expression (*5*). Consistent with this important role, mutations in PcG genes are associated with defective gene expression, developmental abnormalities, embryonic lethality, and cancer (*6*–*8*).

Two general models of epigenetic inheritance have been proposed. In the first model, TFNs form trans-acting positive feedback loops that maintain their own expression and concomitantly direct the expression of other cell type–specific genes (*4*, *9*, *10*). In this model, Polycomb plays a passive role as a default mechanism that silences those genes that are not targeted for activation by cell type–specific TFNs or general transcription factors (*11*–*13*). In the second model, Polycomb plays an active role in inheritance of gene expression programs by forming heritable silent domains that regulate the expression of TFN components and other genes (*2*, *5*, *13*). In this model, positive feedback associated with histone modification and binding activities of Polycomb complexes maintains the modifications and the silent state in cis during cell division. In support of this model, it has previously been shown that in a human cell line, mouse embryonic stem cells, and Chinese hamster ovarian cells, Polycomb silencing induced at a reporter gene inserted in a gene desert can be maintained in the absence of the initiator (*14*–*16*). However, whether Polycomb silencing can be inherited when induced near genes, where it may be influenced by locally bound transcription factors or chromatin modifications, remains unknown.

The PcG genes were identified based on mutations that give rise to homeotic transformations in *Drosophila* (*17*–*19*). Subsequent studies showed that PcG proteins are highly conserved and form multiple Polycomb repressive complexes (PRCs) that have histone modifying and binding activities (*5*). In mammals, canonical PRC1 (cPRC1) is composed of a heterodimer of Polycomb group RING finger protein (PCGF) 2 or 4 with the RING1A or RING1B E3 ubiquitin ligases, which mono-ubiquitinate histone H2AK119, and two additional subunits, CBX2, 4, 6, 7, or 8 chromobox proteins and Polyhomeotic-like (PHC) 1, 2, or 3 (*20*–*22*). The PRC2 complex contains EED, SUZ12, RB binding protein (RBBP) 4 or 7, and the Enhancer of zest 1 (EZH) 1 or 2 histone H3 lysine 27 (H3K27) methyltransferases (*23*–*26*). In addition, variant PRC1 (vPRC1) complexes have been identified that lack CBX subunits and contain RYBP or YAF2 and one of four PCGF proteins (PCGF1, 3, 5, or 6) (*27*).

Polycomb complexes contain both catalytic (writer) and substrate recognition (reader) subunits that mediate extensive cross-talk between them (*28*–*30*). The EED subunit of PRC2, with its seven tryptophan-aspartic acid (WD) 40 repeat domains, binds H3K27me3 and allosterically activates the EZH2 methyltransferase (*28*, *31*–*33*). H3K27me3 is also recognized by the CBX subunits of PRC1, which mediate chromatin compaction and silencing (*34*–*36*). H2AK119ub1 is recognized by the RYBP/YAF2 subunits of vPRC1 (*37*–*39*) in addition to the Jumonji AT rich interactive domain (JARID) 2 and AE binding protein (AEBP) 2 accessory subunits of PRC2 (*40*, *41*). H2AK119ub1 plays a key role in mammalian Polycomb silencing. Deletion of RING1A and RING1B resulting in the loss of H2K119ub1 leads to the concomitant loss of most H3K27me3 domains in mouse embryonic stem cells (*42*, *43*). In addition, the two modification systems perform mutually dependent and independent silencing functions in mouse zygotes and early embryos (*44*, *45*) and interact with accessory factors that bind to unmethylated CpG islands (CGIs) (*46*–*49*). Despite its critical role, it remains unclear whether H2AK119ub1-mediated silencing in the absence of H3K27me3, observed in mouse embryonic stem cells (*42*, *43*, *50*), early mouse and zebrafish embryos (*44*, *45*, *51*), and human embryonic kidney (HEK) 293 cells (*52*) represents an epigenetically heritable silencing modification.

In this study, we used an inducible reporter gene silencing system to examine the role of genomic context and other requirements for Polycomb inheritance. We show that although Polycomb silencing can be established near both ubiquitously expressed housekeeping genes and active developmental genes, it is only heritable at the latter loci. Unexpectedly, aromatic cage mutations that abolish the ability of EED to recognize H3K27me3, and lead to loss of H3K27me3, weaken but do not abolish inheritance. H3K27me3-independent inheritance requires an intact PRC2 complex and mutations that impair the ability of RYBP to recognize H2AK119ub1 lead to its complete loss. Our findings suggest that the PRC1 and PRC2 complexes can interact with each other independently of histone modifications and provide an explanation for how PRC2 can contribute to maintenance of Polycomb silencing independently of H3K27me3.

## RESULTS

### Inheritance of Polycomb silencing is locus dependent

**Fig. 1.**
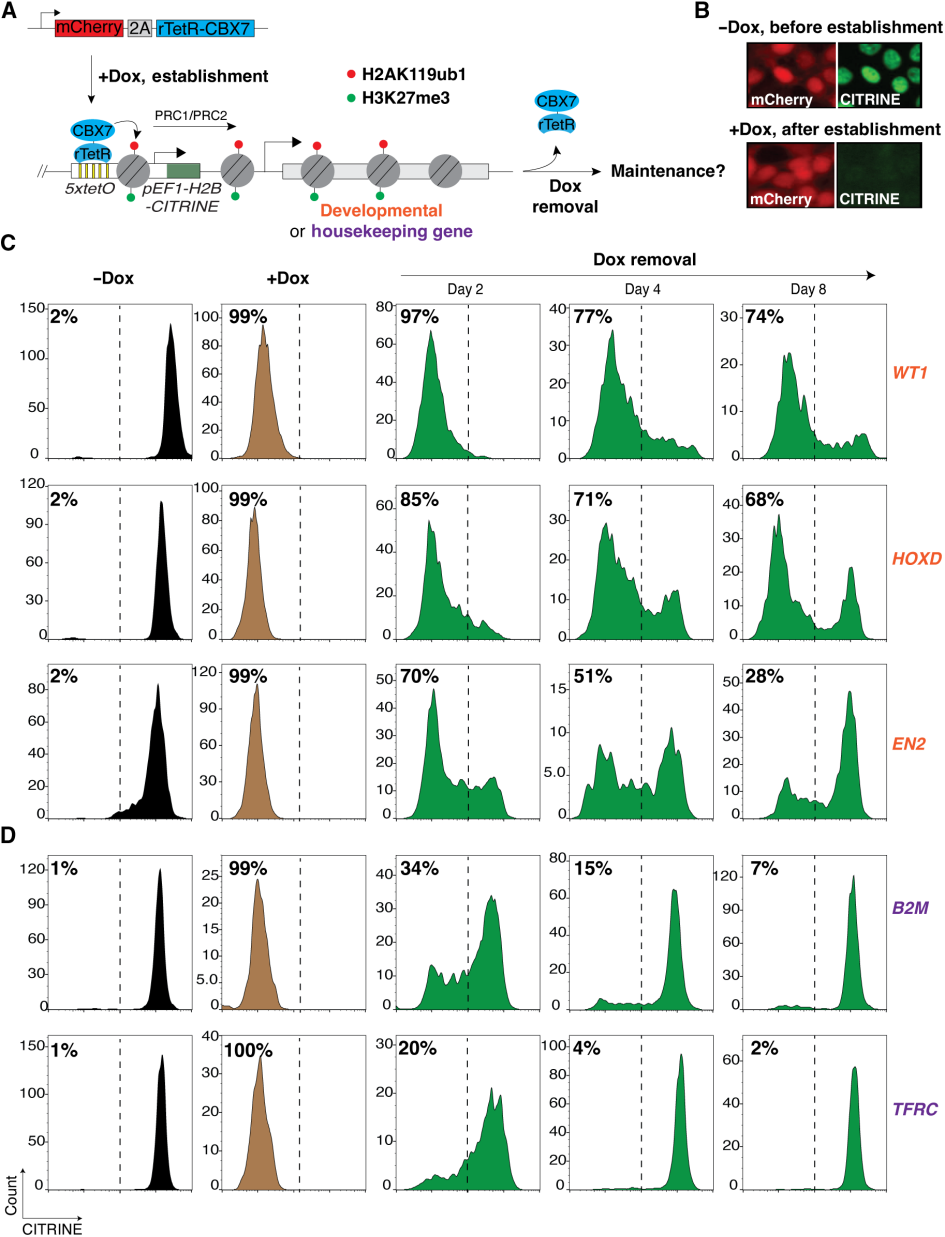
Newly established Polycomb silencing near developmental genes, but not at ubiquitously expressed genes, is heritable. (**A**) Schematic diagram illustrating the strategy for inducible Polycomb silencing by expression of rTetR-CBX7 as a fusion with mCherry-2A in cells carrying the *5xtetO-CITRINE* reporter at various developmental and ubiquitously expressed genes. See figs. S1 and S2 for exact coordinates of reporter insertion. (**B**) Representative fluorescence images showing mCherry and CITRINE expression before and after establishment of silencing (−Dox and +Dox, respectively). (**C**) Flow cytometry histograms showing CITRINE expression before and after establishment of silencing with doxycycline addition (−Dox and +Dox) and at the indicated days after removal of doxycycline (Dox removal) at developmental genes *WT1*, *HOXD*, and *EN2*. Percentages (%) indicate the fraction of CITRINE negative cells. (**D**) Same as (C) but with the reporter inserted near ubiquitously expressed genes *B2M* and *TFRC*.

To investigate the role of DNA context in inheritance of Polycomb silencing, we induced Polycomb silencing at two types of genes, developmentally regulated versus ubiquitously expressed housekeeping genes in HEK293 cells. We chose developmentally regulated genes that are targeted for silencing by Polycomb in human embryonic stem cells (hESCs) but are active in HEK293FT cells. Housekeeping genes are not targeted by Polycomb and are active in most cell types. We integrated a reporter cassette with 5 tetracycline operators (*5xtetO*) upstream of a minimal *EF1* promoter driving *H2B-CITRINE* expression (*5xtetO-H2B-CITRINE*) a few kilobases upstream of multiple Polycomb and non-Polycomb target loci in HEK293FT cells (Fig. 1A). In the same cell lines, we also expressed a reverse Tet repressor (rTetR and Tet-ON) protein fused to the CBX7 subunit of cPRC1 to initiate silencing (Fig. 1A). Recruitment of TetR-CBX7 to *tetO* sites has previously been shown to silence transcription of a reporter gene driven by the minimal EF1 promoter in a Polycomb-dependent manner (*16*). The rTetR-CBX7 protein only binds the *5xtetO* array in the presence of doxycycline (+Dox) and is released upon removal of doxycycline (Dox removal) from the culture medium, allowing us to control the association of the initiator with DNA and assess silencing, H3K27me3, and H2AK119ub1 with and without DNA sequence–dependent initiation (Fig. 1A).

At the Polycomb target loci *WT1*, *EN2*, *HOXD*, and *HOXB*, which encode developmentally regulated transcription factors (Wilms tumor, Engrailed 2, and homeobox transcription factors, respectively), we inserted the *5xtetO-H2B-CITRINE* reporter 3 to 4.5 kb upstream of endogenous promoter regions (Fig. 1A and fig. S1, A to D) (hereafter designated as *GENENAME:CITRINE*). In HEK293FT cells, these genes are devoid of H3K27me3 and are expressed, while in hESCs, they are associated with H3K27me3 and are not expressed (fig. S1, A to D). We cultured cells in +Dox medium to establish silencing for 8 days (~8 cell divisions), with cells in doxycycline-free (−Dox) medium serving as controls (Fig. 1B). Fluorescent-activated cell sorting (FACS) analysis showed that the *WT1:CITRINE*, *EN2:CITRINE*, and *HOXD:CITRINE* reporters were silenced in >95% of cells grown in +Dox medium but were fully expressed in cells grown in −Dox medium in which rTetR-CBX7 does not bind the *5xtetO* sites (Fig. 1C). The *HOXB:CITRINE* reporter was poorly silenced and was not further pursued (fig. S1E). Following establishment, doxycycline was removed to release the rTetR-CBX7 from the *5xtetO* site (Dox removal). As shown in Fig. 1C, silencing of the *WT1:CITRINE* and *HOXD:CITRINE* reporters was maintained at 2, 4, and 8 days after release of the rTetR-CBX7 initiator, with >70% of the *WT1:CITRINE* cells maintaining silencing at day 8 after release. Silencing was also maintained at the *EN2:CITRINE* locus but displayed more rapid decay kinetics (Fig. 1C). At the *WT1* locus, 40 days after release of rTetR-CBX7, 27% of the cells still maintained silencing (fig. S1F). We further verified the inheritance of silencing at the *WT1* locus by CITRINE fluorescence imaging at 2, 4, and 8 days after Dox removal (fig. S1G). These results indicate that transient rTetR-CBX7 recruitment near developmental genes leads to heritable silencing but with decay rates that vary depending on the locus.

As controls, tethering rTetR alone did not lead to silencing of the *WT1:CITRINE* reporter, indicating that silencing was not caused by rTetR-mediated steric inhibition (fig. S1H). Moreover, silencing was maintained when we deleted rTetR-CBX7 8 days after the establishment of silencing, demonstrating that leaky binding of rTetR-CBX7 was not responsible for epigenetic inheritance of silencing at the *WT1* locus (fig. S1I).

We next inserted the *5xtetO-H2B-CITRINE* reporter at the endogenous promoter regions of two ubiquitously expressed housekeeping genes: 4.7 kb upstream of β-2-microglobulin, *B2M*, or 1 kb upstream of the transferrin receptor, *TFRC*. Ubiquitously expressed genes are regulated by general transcription factors, are expressed in all cell types, and are devoid of Polycomb modifications (*53*). In HEK293FT cells, H3K27me3 and H2AK119ub1 are absent at the *B2M* and *TFRC* genes (fig. S2, A and B). We carried out establishment and maintenance assays as we did for the Polycomb target genes. Silencing was robustly established at both *B2M:CITRINE* and *TFRC:CITRINE* reporters (silenced in >95% of cells; Fig. 1D). However, relative to the developmentally regulated genes (Fig. 1C), reporter gene silencing was rapidly lost at these loci (Fig. 1D). By 8 days after the release of rTetR-CBX7, only 7% of the *B2M:CITRINE* reporter and 2% of the *TFRC*:*CITRINE* reporter maintained silencing, which is in the range of background CITRINE negative cells in these assays (Fig. 1D and fig. S2C). These results indicate that Polycomb-mediated silencing established near ubiquitously expressed genes, *TFRC* and *B2M,* is rapidly reversed in the absence of continuous initiation and is not inherited.

To verify rTetR-CBX7 binding and release, we constructed rTetR-CBX7-Flag cell lines, which expressed similar levels of rTetR-CBX7-Flag and displayed heritable silencing at the *WT1* but not the *TFRC* locus (fig. S3, A and B). Experiments using these cell lines showed that rTetR-CBX7 localized with similar efficiency to the *WT1:CITRINE* and *TFRC:CITRINE* loci under establishment conditions (fig. S3C). At the *TFRC:CITRINE* reporter, no rTetR-CBX7 binding was detected 4 days after its release by growth in medium lacking Dox (fig. S3C, right). At the *WT1:CITRINE* reporter, there was binding of the rTetR-CBX7 under maintenance conditions but at lower levels than in the establishment phase (fig. S3C, left). rTetR-CBX7 would be expected to be recruited to the locus during the maintenance phase via the interaction of rTetR-PRC1 with Polycomb histone modifications, independently of the *5xtetO* array. As expected, in the absence of establishment (cells grown in −Dox medium), rTetR-CBX7 did not bind to the reporter locus, as the chromatin immunoprecipitation–quantitative polymerase chain reaction (ChIP-qPCR) signals were similar to the cell line where rTetR-CBX7 was absent (fig. S3C). This result further confirmed that there was no leaky binding of rTetR-CBX7 to the 5x*tetO* array in the absence of Dox.

### Heritable silencing is associated with inheritance of Polycomb modifications

**Fig. 2.**
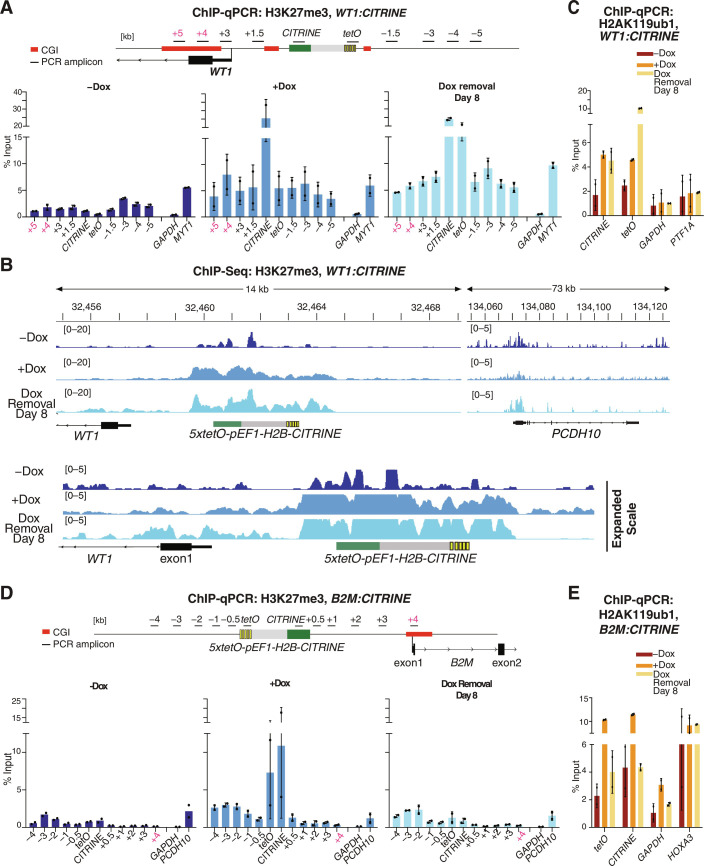
Memory of newly established H3K27me3 and H2AK119ub1 near or at the developmental gene, *WT1*, but not near the ubiquitously expressed gene, *B2M*. (**A**) ChIP-qPCR analysis of H3K27me3 enrichment at the reporter locus and surrounding regions at the *WT1* locus before establishment (−Dox), after establishment (+Dox), and during the maintenance phase (Dox removal). *GAPDH* served as a negative control and the native Polycomb-repressed *MYT1* gene as a positive control. Results for two biological replicates are presented. Error bars represent SDs. The location of PCR amplicons and CGIs are highlighted in the map at the top. (**B**) Genome browser snapshots of H3K27me3 ChIP-seq reads at the reporter locus inserted near *WT1* and the endogenous Polycomb-silenced *PCDH10* genes. Expanded scale (bottom) highlights the spreading of H3K27me3 to the promoter of WT1 and its maintenance. (**C**) Same as (A) but H2AK119ub1 ChIP-qPCR. (**D**) ChIP-qPCR analysis of H3K27me3 enrichment at the reporter locus inserted near *B2M* and surrounding regions before establishment (−Dox), after establishment (+Dox), and during the epigenetic maintenance of silencing (Dox removal). *GAPDH* served as a negative control and the native Polycomb-repressed *PCDH10* gene as a positive control. Results for two biological replicates are presented. Error bars represent SDs. (**E**) Same as (D) but H2AK119ub1 ChIP-qPCR; *HOXA3* served as a positive control.

We next examined the establishment and maintenance of Polycomb-associated histone modifications at the developmental *WT1* and housekeeping *B2M* and *TFRC* loci. At the *WT1:CITRINE* locus, ChIP-qPCR and ChIP sequencing (ChIP-seq) results showed that a domain of H3K27me3 and H2AK119ub1 was established by rTetR-CBX7 and, consistent with maintenance of *WT1:CITRINE* silencing, these modifications were maintained 8 days after the release of rTetR-CBX7 (Fig. 2, A to C, and fig. S4A). The H3K27me3 domain extended to ~7 kb on both sides of the 5x*tetO* sites and spread to the promoter and first exon of the *WT1* gene and was enriched at levels comparable to an endogenous Polycomb target gene, *MYT1* (Fig. 2, A and B, see expanded scale in Fig. 2B). As expected, rTetR-CBX7–mediated H2A119ub1 and H3K27me3 were accompanied by the recruitment vPRC1 subunits PCGF1, RYBP, and Lysine demethylase 2B (KDM2B), and to a lesser extent, the CBX2 subunit of cPRC1 (fig. S4F). The weaker CBX2 recruitment may be due to competition for H3K27me3 nucleosomes with rTetR-CBX7 and other cPRC1 chromobox family members. The newly established H3K27me3 and H2AK119ub1 spanning the endogenous *WT1* sequences were maintained 8 days after of the release of rTetR-CBX7 (Fig. 2, A to C, and fig. S4A, highlighted in red), indicating that the establishment and epigenetic inheritance of the Polycomb domain were not restricted to the reporter gene. At the *B2M* and *TFRC* housekeeping loci, H3K27me3 and H2AK119ub1 were enriched at regions surrounding the reporter cassette but were lost in the maintenance phase, 8 days after the release of rTetR-CBX7 (Fig. 2, D and E, and fig. S4, B to E). In contrast to the *WT1* locus, H3K27me3 and H2AK119ub1 did not spread to the promoter region of either *B2M* or *TFRC* genes (Fig. 2, D and E, and fig. S4, B to E). Therefore, the rTetR-mediated establishment of silencing is coupled to histone H3K27me3 and H2AK119ub1. However, the modifications can be epigenetically maintained at the *WT1* but not the *B2M* or *TFRC* genes.

### H3K27me3 recognition by PRC2 is not required for short-term inheritance

**Fig. 3.**
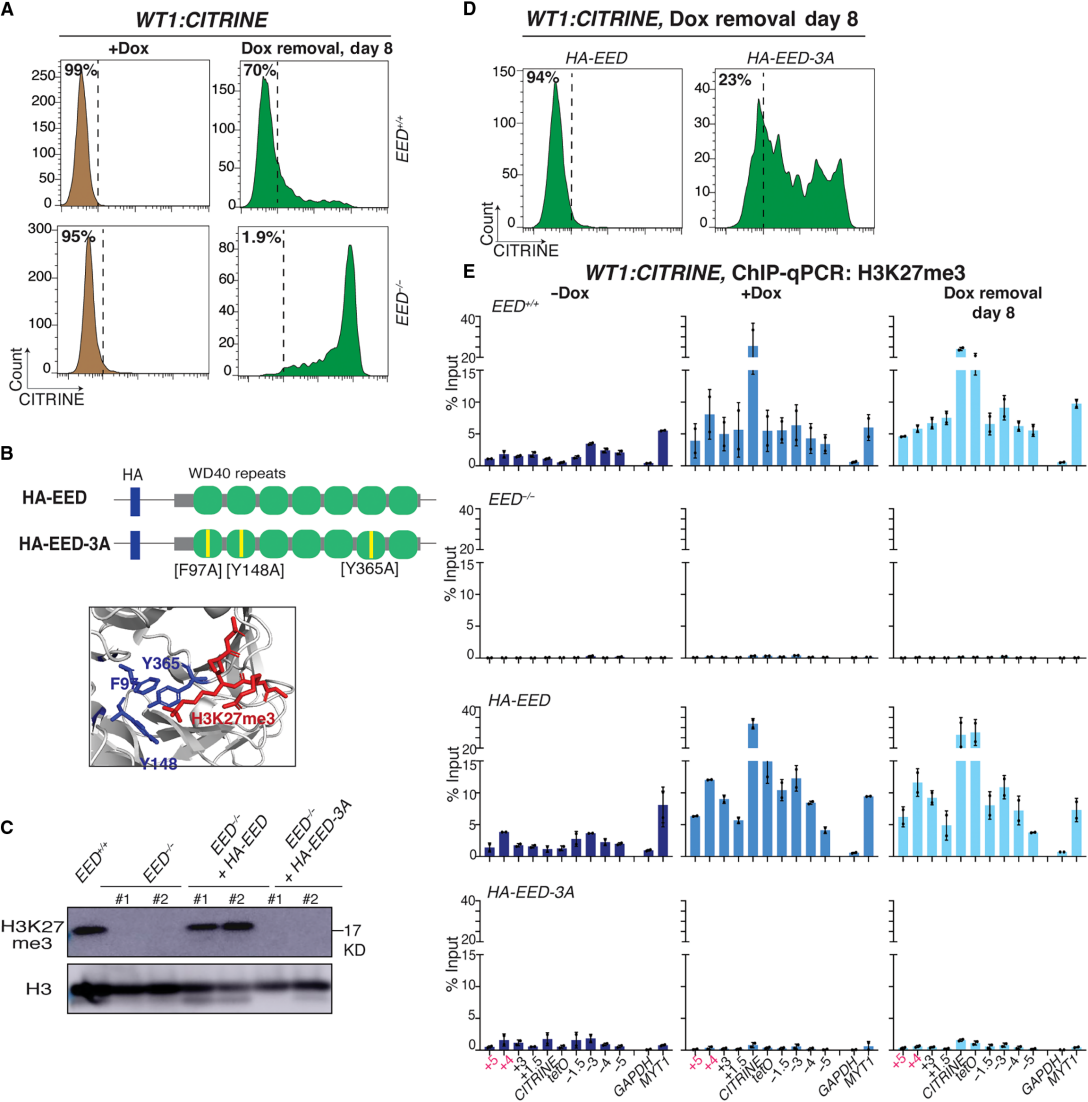
H3K27me3 recognition by PRC2-EED is partly dispensable for epigenetic inheritance of Polycomb silencing. (**A**) Flow cytometry histograms showing CITRINE expression, with the reporter inserted at the Polycomb target locus, *WT1*, after establishment of silencing (+Dox) and 8 days after removal of doxycycline (Dox removal) in control (*EED^+/+^*) and *EED*-deleted cell line (*EED^−/−^*). (**B**) Schematic diagram showing the wild-type EED (*HA-EED*) and the three residues mutated [F97A, Y148A, and Y365A] in the aromatic cage of EED (*HA-EED-3A*) (top). A snapshot of the structure of *EED* aromatic cage with bound H3K27me3 peptide. The *EED* is colored in gray, the F97, Y148, and Y365 residues are in blue, and the H3K27me3 peptide is red (bottom) (PBD3IIW) (*28*). (**C**) Western blot showing H3K27me3 levels in *EED^+/+^*, *EED^−/−^*, and *EED^−/−^* overexpressing *HA-EED* or *HA-EED-3A.* Histone H3 is used as a loading control. (**D**) Flow cytometry histograms showing CITRINE expression 8 days after removal of doxycycline (Dox removal) in *HA-EED* and *HA-EED-3A* cells. (**E**) ChIP-qPCR analysis of H3K27me3 enrichment at the reporter locus and surrounding regions before establishment (−Dox), after establishment (+Dox), and 8 days after removal of doxycycline (Dox removal) in *EED^+/+^*, *EED^−/−^*, *HA-EED*, and *HA-EED-3A* cells*. GAPDH* served as a negative control and the native Polycomb-repressed *MYT1* gene as a positive control. Error bars represent SDs.

We next tested the requirement for the PRC2 read-write activity in epigenetic inheritance of Polycomb silencing at the *WT1:CITRINE* locus. As expected (*33*), in *EED^−/−^* knockout *WT1:CITRINE* reporter cell lines, silencing was efficiently established (in +Dox medium), but maintenance of silencing was abolished 8 days after Dox removal (Fig. 3A). Similarly, knockout of the SUZ12 subunit, a scaffold protein that is required for PRC2 integrity (*54*), or double knockout of H3K27 methyltransferase EZH1 and EZH2, abolished the maintenance of *WT1:CITRINE* silencing (fig. S5, A to E). We then attempted to rescue the maintenance defect of *EED^−/−^* cells with either wild-type *HA-EED* or an aromatic cage mutant (F97A, Y148A, and Y365A; referred to as *HA-EED-3A*), which does not bind to H3K27me3 and thus cannot allosterically activate EZH2 (*28*, *33*) (Fig. 3, B and C). Western blotting showed that HA-EED and HA-EED-3A were expressed to similar levels (fig. S6A). Consistent with previous results (*26*), H3K27me3 was lost in *EED^−/−^* cells and was rescued in cells transfected with wild-type *HA-EED* but not the mutant *HA-EED-3A* (Fig. 3C). The expression of wild-type HA-EED fully rescued the maintenance defect of the *CITRINE* reporter in the *EED^−/−^* cells (Fig. 3D). Unexpectedly, expression of the HA-EED-3A mutant restored inheritance in a substantial fraction of cells (~23%) 8 days after the release of rTetR-CBX7 (Fig. 3D). Since HA-EED-3A cannot bind to H3K27me3, we conclude that H3K27me3 recognition by EED/PRC2 is not required for transient inheritance of *WT1:CITRINE* silencing.

To test the possibility that low levels of H3K27me3, which may not have been detected in a whole-cell lysate Western blot (Fig. 3C), were present at the *CITRINE:WT1* locus, we performed ChIP-qPCR and ChIP-seq for H3K27me3. H3K27me3 was restored at the *WT1* locus during establishment and maintenance in wild-type *HA-EED* cells but was close to background levels in *HA-EED-3A* or *EED^−/−^* cells (Fig. 3E and fig. S6B, left). Similarly, at the endogenous *PCDH10* Polycomb target gene, H3K27me3 enrichment was restored in *HA-EED* cells but not *HA-EED-3A* cells (fig. S6B, right). Consistent with the idea that PRC2 can play a noncatalytic role in inheritance of *WT:CITRINE* silencing, we found that the deletion of both *EZH1* and *EZH2* or the AEBP2 accessory subunit of PRC2 in HA-EED-3A cells abolished the residual inheritance of *WT1:CITRINE* silencing (fig. S6, C to F). Together, these results indicate that H3K27me3 and PRC2 read-write capability contribute to the stable maintenance of Polycomb-mediated silencing, but short-term epigenetic maintenance can occur in the absence of detectable H3K27me3. PRC2 therefore contribute to the inheritance of Polycomb silencing via both catalytic and noncatalytic mechanisms.

### Recognition of CG-rich DNA by MTF2-PRC2 is required for epigenetic inheritance

**Fig. 4.**
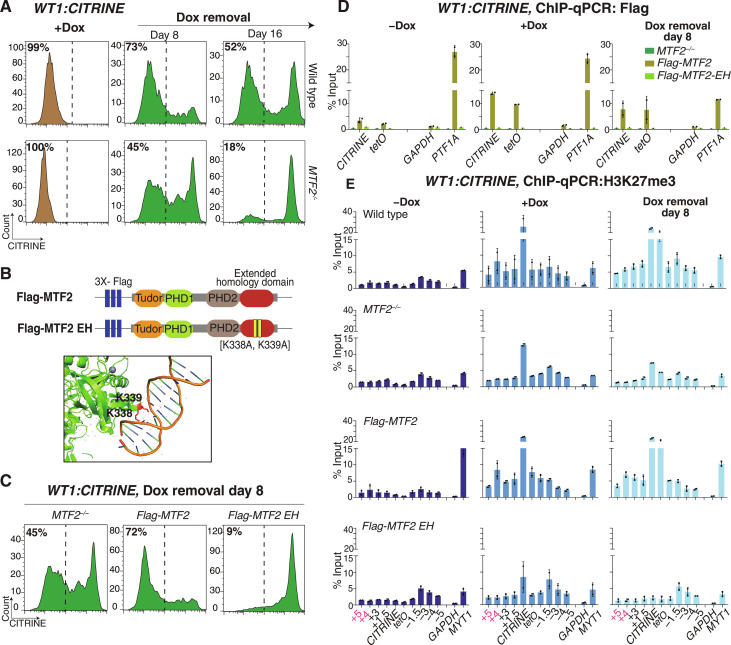
The DNA binding activity of *MTF2* is required for epigenetic inheritance of Polycomb silencing at the *WT1* developmental gene. (**A**) Flow cytometry histograms showing CITRINE expression, inserted at the Polycomb target locus, *WT1*, after establishment of silencing (+Dox) and at the indicated days after removal of doxycycline (Dox removal) in wild-type control (WT) and *MTF2*-deleted cell line (*MTF2^−/−^)*. (**B**) Schematic diagram showing the wild-type MTF2 (*Flag-MTF2*) and the two residue mutations [K338A and K339A] generated in the extended homology (EH) domain of MTF2 (*Flag-MTF2-EH*) (top). A snapshot of the structure of *MTF2* EH domain with bound DNA. MTF2 is colored in green, the K338 and K339 residues are in red, and the DNA is multicolored (PDB: 5XFR) (*47*). (**C**) Flow cytometry histograms showing CITRINE expression at day 8 after removal of doxycycline (Dox removal) in *MTF2^−/−^* and *MTF2^−/−^* cells overexpressing Flag-MTF2 or Flag-MTF2-EH*.* (**D**) ChIP-qPCR analysis of Flag enrichment at the reporter locus and surrounding regions before establishment (−Dox), after establishment (+Dox), and 8 days after removal of doxycycline (Dox removal) in *MTF2^−/−^* and *MTF2^−/−^* overexpressing Flag-MTF2 and Flag-MTF2-EH. *GAPDH* served as a negative control and the native Polycomb-repressed *PTF1A* gene as a positive control. Error bars represent SDs. (**E**) Same as (D) but ChIP for H3K27me3 in WT, *MTF2^−/−^* and *MTF2^−/−^* overexpressing Flag-MTF2 or Flag-MTF2-EH. (**F** and **G**) Same as (D) but ChIP for MTF2 at the *WT1* (F) and *TFRC* (G) loci.

The core PRC2 complex associates with accessory subunits that bind CG-rich DNA at Polycomb target genes (*46*–*48*), including association with PCL1–3 proteins to form PRC2.1 and with JARID2 to form PRC2.2 (*55*–*57*). In addition, developmental genes that are targeted for Polycomb silencing have been noted to contain a higher density of CGIs (*46*, *58*, *59*) and, relative to ubiquitously expressed housekeeping genes, the developmental genes studies here all had more annotated CGIs that extended beyond the promoter-associated CGI present at the ubiquitously expressed genes (fig. S7A). Since intact PRC2 was required for H3K27me3-independent inheritance (figs. S5 and S6, C to F), we used knockout and rescue experiments to investigate the possible role of PRC2 accessory proteins and their CGI-binding domains in inheritance of silencing at the *WT1:CITRINE* locus. Deletion of *PCL2/MTF2* (*MTF2^−/−^*), confirmed by Western blotting (fig. S7B), showed strong loss of *WT1:CITRINE* silencing by 8 days after release of the rTetR-CBX7 and near-complete loss by 16 days after release (Fig. 4A), indicating that MTF2 was required to maintain silencing at the *WT1* locus.

MTF2 binds to CG-rich DNA through its extended homology domain (*46*, *47*). To determine whether the DNA binding activity of MTF2 was necessary for the maintenance of the *WT1:CITRINE* silencing, we introduced 3x-Flag-tagged wild-type MTF2 (Flag-MTF2) or mutant extended homology domain MTF2 (K338A and K339A; referred to as Flag-MTF2-EH), which does not bind DNA in vitro, into *MTF2^−/−^* cells (Fig. 4B). Western blotting showed that the wild-type and mutant MTF2 proteins were expressed at similar levels (fig. S7C). In *MTF2^−/−^* cells overexpressing wild-type Flag-MTF2, maintenance of *CITRINE* reporter silencing was restored, while in *MTF2^−/−^* cells overexpressing the Flag-MTF2-EH mutant, maintenance was not restored (Fig. 4C). ChIP-qPCR and ChIP-Seq for *Flag-MTF2* rescue cells demonstrated that wild-type Flag-MTF2 was recruited during establishment and maintenance (Fig. 4D and fig. S7D). Notably, Flag-MTF2-EH was not recruited to either the reporter locus or the endogenous *PTF1* and *FOXQ1* Polycomb target genes (*47*), during either establishment or maintenance phases (Fig. 4D and fig. S7D). These observations suggest that the stable binding of MTF2/PRC2 to the *CITRINE* reporter locus requires the interaction of MTF2 with DNA, even in the presence of the bound rTetR-CBX7 initiator. In addition, ChIP-qPCR and ChIP-Seq in *MTF2^−/−^* showed reduced H3K27me3 levels during both establishment and maintenance at the *WT1:CITRINE* reporter, which were restored in wild-type *Flag-MTF2* but not in *Flag-MTF2-EH* cells during maintenance (Fig. 4E and fig. S7, D and E). *Flag-MTF2-EH* cells also had reduced H3K27me3 levels at the endogenous Polycomb target genes *MYT1* and *PCDH10*, and its localization at *FOXQ1* was abolished (Fig. 4E and fig. S7, D and E). These results demonstrate that the DNA binding activity of MTF2 is required for its efficient recruitment by rTetR-CBX7 and for maintenance of Polycomb silencing at the *WT1* locus and show that the *WT1* locus has similar requirements for MTF2 as endogenous Polycomb targets.

We also deleted three other DNA binding proteins, PRC2.2 subunit JARID2 and PRC2.1 subunits PHF1 (PCL1) and PHF19 (PCL3) in the cell line containing the *WT1:CITRINE* reporter. We found that deletion of *JARID2* and *PHF19* had no effect on establishment or maintenance (fig. S8, A to D), but the deletion of *PHF1* led to slow loss in maintenance by day 16 after rTetR-CBX7 release, similar to *MTF2^−/−^* cells (fig. S8, E and F). These results suggest that other PRC2 accessory proteins also contribute to H3K27me3-independent maintenance of silencing.

### H2AK119ub1 and vPRC1 can promote heritable short-term silencing independently of H3K27me3

**Fig. 5.**
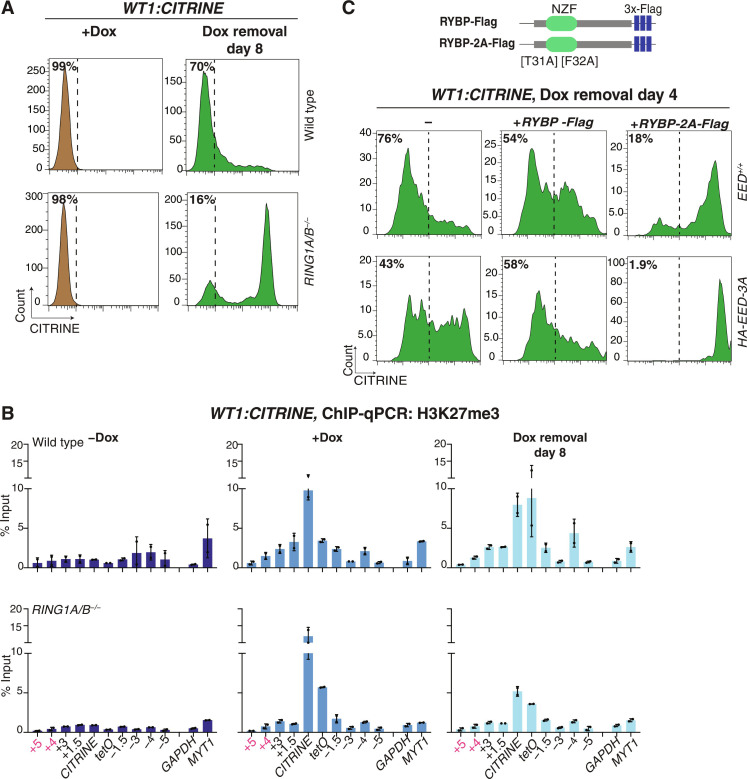
vPRC1 read-write and H2AK119ub1 contribute to epigenetic inheritance of Polycomb silencing independently of H3K27me3 recognition by PRC2. (**A**) Flow cytometry histograms showing CITRINE expression after establishment of silencing (+Dox) and 8 days after removal of doxycycline (Dox removal) in wild-type (WT) and *RING1A/B*-deleted cell line (*RING1A/B^−/−^)* with the reporter inserted at the Polycomb target locus, *WT1.* (**B**) ChIP-qPCR analysis of H3K27me3 enrichment at the reporter locus and surrounding regions before establishment (−Dox), after establishment (+Dox), and 8 days after removal of doxycycline (Dox removal) in control and *RING1A/B^−/−^* cells. *GAPDH* served as a negative control and the native Polycomb-repressed *MYT1* gene as a positive control. Error bars represent SDs. (**C**) Schematic diagram showing the wild-type RYBP (*RYBP-Flag*) and the two residues mutated [T31A and F32A] generated in the ubiquitin binding domain of RYBP (*RYBP-2A-Flag*) (top). Flow cytometry histograms showing CITRINE expression 4 days after removal of doxycycline (Dox removal) in *EED^+/+^* and *HA-EED-3A* cells overexpressing RYBP-Flag or RYBP-2A-Flag. (−) refers to nontransfected cells (bottom).

We next tested the possibility that H3K27me3-independent inheritance of Polycomb silencing is mediated by vPRC1 read-write activity (*37*). Consistent with previous findings, deletion of *RING1A* and *RING1B* (*RING1A/B^−/−^)* abolished H2AK119ub1 in our *WT1* reporter cell line (fig. S9, A to C). The establishment of *CITRINE* reporter silencing was unaffected in *RING1A/B^−/−^* cells, but the maintenance of silencing was greatly diminished (Fig. 5A). Silencing persisted in a small fraction (16%) of *RING1A/B^−/−^* cells, suggesting that some inheritance could occur independently of H2AK119ub1 (Fig. 5A). ChIP-qPCR and ChIP-Seq of H3K27me3 showed that during establishment, H3K27me3 was deposited in both RING1A/B^+/+^ and *RING1A/B^−/−^* cells but was greatly decreased by 8 days after release of the rTetR-CBX7 concomitant with de-repression of the *WT1:CITRINE* reporter in *RING1A/B^−/−^* cells (Fig. 5B and fig. S9D). These results suggest that rTetR-CBX7 can recruit PRC2 and H3K27me3 independently of H2AK119ub1. In addition, they indicate that in the absence of H2AK119ub1, H3K27me3 is likely to recruit downstream factors that silence the reporter gene, but it cannot maintain the silent state in most cells.

The PRC2 complex containing the mutant HA-EED-3A may promote maintenance of silencing by helping propagate PRC1-mediated H2AK119ub1. In support of this hypothesis, during both the establishment (+Dox) and maintenance (Dox removal) phases, H2AK119ub1 was enriched to similar levels at the *WT1:CITRINE* reporter locus and the endogenous HOXA10 gene in *HA-EED-3A* and *EED^+/+^* cells (fig. S9E). H2AK119ub1 is recognized by the RYBP/YAF2 subunit of vPRC1, which allosterically activates RING1A/B ubiquitination activity (*37*, *38*). To test whether this H2AK119ub1 read-write contributes to the inheritance of silencing, we overexpressed either wild-type RYBP-Flag or a ubiquitin binding mutant RYBP (T31A and F32A, called RYBP-2A-Flag) (*37*, *39*) in *EED^+/+^* and *HA-EED-3A* in the *WT1:CITRINE* reporter cells (Fig. 5C). Western blotting showed that the wild-type RYBP-Flag and mutant RYBP-2A-Flag were expressed at similar levels (fig. S9F). We reasoned that the mutant RYBP may behave as a dominant negative by competing with wild-type RYBP for incorporation into vPRC1. Overexpression of RYBP-2A-Flag, but not wild-type RYBP-Flag, greatly reduced the maintenance of *WT1:CITRINE* silencing in *EED^+/+^* cells and abolished the partial maintenance phenotype of *HA-EED-3A* cells (Fig. 5C). These results demonstrate that the H2AK119ub1 read-write capability of vPRC1 contributes to epigenetic maintenance of Polycomb silencing independently of H3K27me3. They further uncover a role for EED/PRC2 in this inheritance that is independent of H3K27me3 and its recognition.

### Polycomb complexes physically interact

**Fig. 6.**
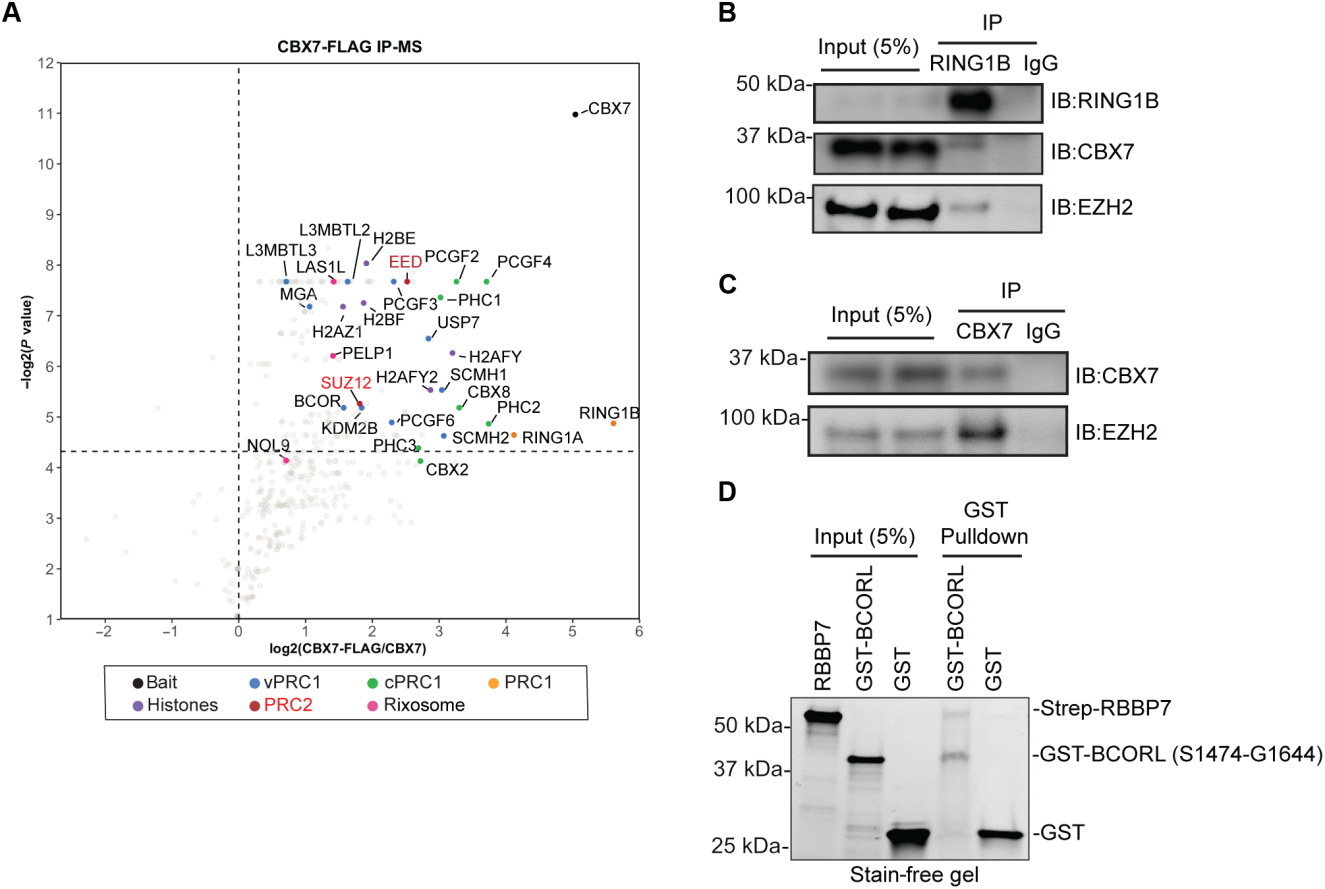
Interactions between Polycomb complexes. (**A**) Volcano plot displaying the results of MS identification of proteins enriched in Flag immunoprecipitations from cells expressing rTetR-CBX7-3xFlag relative to untagged cells from two independent experiments. Subunits of the cPRC1, vPRC1, PRC2, rixosome, and histones are highlighted. (**B**) Coimmunoprecipitations showing the association of RING1B with PRC1 subunit CBX7 and PRC2 subunits EZH2 in HEK 293FT cells. IB, immunoblot. (**C**) Coimmunoprecipitations showing the association of CBX7 and PRC2 subunits EZH2 in HEK 293FT cells. (**D**) Pulldown assays using bead-immobilized GST or GST-BCORL(S1474-G1644) proteins and Strep-RBBP7, showing binding of Strep-RBBP7 to the GST-BCORL fragment.

The observation that rTetR-CBX7 initiated H3K27me3 and silencing in RING1A/B^−/−^ cells suggests that CBX7/PRC1 can interact with and recruit PRC2 in the absence of H2AK119 ubiquitination (Fig. 5A). To test this hypothesis, we performed rTetR-CBX7-3xFlag immunoprecipitation followed by mass spectrometry (IP-MS). As expected, all known core subunits of the cPRC1 complex, RING1A/B, PHC1/2/3, PCGF2/4, and rixosome subunits (LAS1L and PELP1), were enriched in rTetR-CBX7-3xFlag immunoprecipitations (Fig. 6A and table S10) (*27*, *60*). The EED and SUZ12 subunits of PRC2, were also highly enriched in the rTetR-CBX7-3xFlag immunoprecipitations (Fig. 6A), supporting the hypothesis that PRC1 and PRC2 complexes physically interact. In addition, KDM2B, BCL6 co-repressor (BCOR), BCORL, PCGF1, and PCGF3, which are subunits of vPRC1 complexes (*27*, *49*, *61*, *62*), were enriched in the immunoprecipitations (Fig. 6A), suggesting direct or indirect interactions between cPRC1 and vPRC1 complexes. As an independent verification of the IP-MS results, we found that the CBX7 subunit of cPRC1 and the EZH2 subunit of PRC2 coimmunoprecipitated with RING1B (Fig. 6B) and EZH2 coimmunoprecipitated with CBX7 (Fig. 6C).

PRC1-PRC2 interactions were further supported by an in silico AlphaFold-Multimer (AF-M) screens for pairwise interactions between core subunits of the cPRC1, vPRC1, and PRC2 complexes. We carried out a total of 740 pairwise structural predictions, 51 of which (7%) showed interface-predicted template modeling (ipTM) scores higher than 0.5. This set included interacting partners that were previously identified based on structural or biochemical results and potential new interactions (table S11 and fig. S10). AF-M successfully predicted the structures of each vPRC1, cPRC1, and PRC2 with the highest ipTM score between subunits within each complex (table S11; predicted structures are available in ModelArchive). AF-M screens also predicted interactions between the subunits of vPRC1 and PRC2 and cPRC1 and PRC2, some with high ipTM scores (table S11). Among the highest-ranking interactions, the paralogous RBBP4 and RBBP7 subunits of PRC2 were predicted to interact with the paralogous BCOR and BCORL subunits of vPRC1 (fig. S10, A to E). The interaction of RBBP7 with BCORL involved mainly loops between the WD40 repeats and the extreme C-terminal α helix of RBBP7 (amino acids 417 to 425) and inter-α helical loops of BCORL ankyrin repeats (amino acids 1484 to 1639) (fig. S11, A to C). In addition, AF-M predictions showed that RBBP7 could assemble into the vPRC1 complex (fig. S11, D and E). However, the predicted BCORL-RBBP7 interaction interface clashes with part of the SUZ12-RBBP7 interaction interface, involving amino acids 134 to 144 of SUZ12 (fig. S11, F to H). This region of SUZ12 forms an α helix that contacts the side of the BCORL-interacting RBBP7 β propellers facing away from the core of the PRC2 complex (fig. S11, F to H). Any interaction between the PRC2 and vPRC1 complexes would therefore require a conformational rearrangement of PRC2. Since SUZ12 makes other extensive interactions with RBBP7, it is possible that the 134-144 α helix acts as a gatekeeper that regulates the interaction of RBBP7/PRC2 with BCORL/vPRC1. Consistent with AlphaFold predictions, glutathione *S*-transferase (GST) pulldown experiments showed that GST-BCORL(1474-1644) but not GST pulled down full-length RBB7 (Fig. 6H). Further studies are required to test the validity of the remaining newly predicted interaction interfaces.

## DISCUSSION

**Fig. 7.**
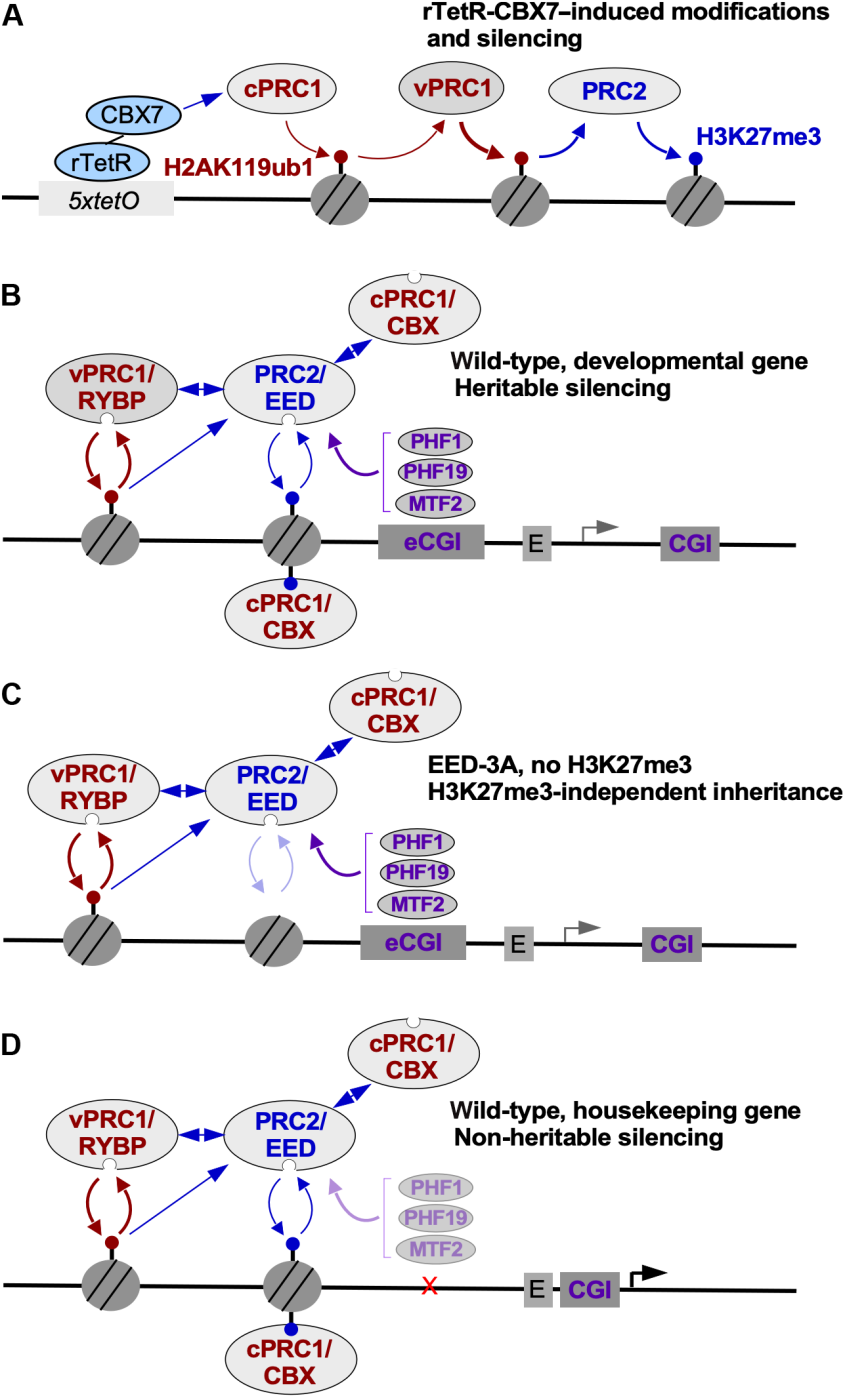
Interactions involving PRCs and histone modifications that mediate heritable silencing. (**A**) Diagram showing the expected rTetR-CBX7-initiated recruitment of PRCs (cPRC1, vPRC1, and PRC2) leading to H2AK119 mono-ubiquitination (H2AK119ub1) and H3K27 trimethylation (H3K27me3). (**B**) Summary of interactions in wild-type cells at a developmental gene targeted by Polycomb. (**C**) In the absence of H3K27me3 in EED-3A cells, intact PRC2 and PRC1 complexes mediate heritable silencing. (**D**) At housekeeping genes, Polycomb silencing is not heritable due to the absence of extended CpG islands (eCGI) and/or other differences between housekeeping genes and developmental genes.

In this study, we show that newly established domains of Polycomb repression at developmental loci, but not near housekeeping genes, can be inherited for many cell divisions. Efficient inheritance requires both PRC1-catalyzed H2AK119ub1 and PRC2-catalyzed H3K27me3 and their associated read-write activities. However, in the absence of H3K27me3, inheritance can still be mediated by PRC1-catalyzed H2AK119ub1. H3K27me3-independent inheritance requires the RYBP-vPRC1 read-write activity and an intact PRC2 complex, suggesting that in addition to its well-established read-write function, PRC2 plays a noncatalytic role in inheritance of Polycomb silencing (Fig. 7). In addition, our findings suggest that CBX7/PRC1 can recruit PRC2 and initiate H3K27 methylation independently of H2AK119ub1.

### Restricted epigenetic inheritance of Polycomb silencing

Our findings demonstrate that the inheritance of Polycomb silencing is DNA context dependent. Once established, Polycomb memory can persist at developmental genes that are regulated by cell type–specific transcription factors. However, Polycomb domains established near transcriptionally active ubiquitously expressed genes are unstable, being lost within two cell divisions after removal of the rTetR-CBX7 initiator. Thus, the inheritance of Polycomb silencing at mammalian genic loci depends on specific sequence features of developmental genes and may be antagonized by constitutively ON enhancers at housekeeping genes. Additional studies are required to understand the sequence features that lead to distinct effects on heritability at developmental versus housekeeping genes. However, these sequence features are likely to involve the higher abundance of CGIs at developmental genes compared to nontarget genes such as ubiquitously expressed genes (*46*, *58*, *59*), as well as the presence or absence of trans-acting cell type–specific transcription factors that target these sequences (*13*). In support of a role for CGIs in heritable Polycomb silencing, we found that point mutations in the PRC2 accessory subunit MTF2 that disrupt its ability to recognize and bind to CG-rich DNA strongly impaired Polycomb inheritance. The importance of MTF2-mediated CGI recognition in inheritance of Polycomb silencing at developmental genes is additionally supported by our observation that, after the establishment of silencing, both MTF2 and H3K27me3 were enriched at the extended CGIs of developmental genes but not the single promoter-associated CGI of the ubiquitously expressed *TFRC* gene.

### Conserved role for DNA sequence in epigenetic inheritance

Specific DNA sequences have also been shown to be required for inheritance of silent chromatin in *Drosophila* and the fission yeast *Schizosaccharomyces pombe* (*63*–*66*). In *Drosophila*, Polycomb Response Elements (PREs) are composite DNA elements that are required for both the establishment and inheritance of H3K27me3 domains and silencing of the *HOX* gene clusters (*67*–*69*). Similarly, in *S. pombe*, composite DNA elements called maintainers are essential for maintenance of H3K9me3 domains unless an antisilencing factor that promotes H3K9me3 demethylation is deleted (*65*, *66*, *70*). Our findings challenge DNA sequence–independent models of Polycomb inheritance based solely on the read-write positive feedback associated with Polycomb complexes and instead suggest that specific DNA sequences, maintainers in *S. pombe*, PREs in *Drosophila*, and extended CGIs or other promoter-associated elements in mammals, act as cis memory modules that serve direct and broadly conserved roles in epigenetic inheritance of different types of silent chromatin. Since the CGI-binding activities of PRC2 are required for inheritance of a newly established silencing, CGIs are likely to promote inheritance by participating in cooperative recruitment of PRC2 (Fig. 7). CGIs may similarly participate in cooperative recruitment of vPRC1, which can bind to H2AK119ub1 via its RYBP subunit and has been shown to interact with a CGI-binding protein KDM2B (*37*–*39*).

### H2AK119ub1-mediated Polycomb inheritance

Our results suggest that while intact PRC2 is required for the inheritance of silencing, the ability of its EED subunit to recognize H3K27me3 and promote H3K27 methylation is partially dispensable (Fig. 7B and fig. S14). In the absence of H3K27me3, we found that PRC1-mediated H2AK119 ubiquitination and the ability of the RYBP subunit of vPRC1 to recognize H2AK119ub1 are essential for heritable silencing. PRC2 plays a noncatalytic role in this transient inheritance since the complete deletion of any of its subunits or accessory factors abolishes inheritance. Our structural predictions suggest that PRC2 interacts directly with vPRC1 through the interaction of its RBBP4/7 subunit with the BCOR/BCORL subunit of vPRC1.1 (fig. S14). In addition, PRC2 can localize to H2AK119ub1 nucleosomes via its accessory subunits JARID2 and AEBP2 (*41*, *71*) and AlphaFold predicts an interaction between the EED subunit of PRC2 and ubiquitin, which remains to be experimentally tested (fig. S13).

H2AK119ub1-dependent and H3K27me3-independent inheritance of silencing raise questions about the mechanism of chromatin inheritance during DNA replication. In the current models for inheritance of parental histones during DNA replication, parental histone H3/H4 tetramers are thought to be transferred to newly synthesized daughter DNA strands. Histone H2A/H2B dimers, on the other hand, are thought to be rapidly exchanged and not inherited (*72*–*74*). Whether H3K27me3-independent inheritance of silencing is mediated through the inheritance of parental H2AK119ub1/H2B or other features of Polycomb domains needs to be investigated. After the completion of this study (*75*), Flury *et al.* (*76*) reported that parental histone H2A/H2B are rapidly recycled during DNA replication. Their findings, which are based on strand-specific ChIP experiments and show that H2A/H2B are transferred to daughter DNA strands shortly after DNA replication, provide independent support for the idea that H2AK119ub1 is inherited during DNA replication and are consistent with our findings showing that H2AK119ub1 can mediate the inheritance of silencing independently of H3K27me3.

A previous study concluded that vPRC1 is not required for inheritance of TetR-CBX7–induced silencing (*16*). However, this conclusion was based on a partial siRNA-mediated knockdown of RYBP and left open the possibility that YAF2, the RYBP paralog, may compensate for the loss of RYBP. In our experiments, we overexpressed a mutant RYBP protein that cannot recognize H2AK119ub1 and would be expected to act as a dominant negative. The loss of inheritance observed using this strategy strongly suggests that vPRC1 is required for maintenance of Polycomb silencing.

### Possible direct cross-talk between Polycomb complexes

The cross-talk between Polycomb complexes is generally thought to be mediated by the ability of each complex to recognize the histone modification that is catalyzed by the other complex. Thus, cPRC1 complexes bind to PRC2-catalyzed H3K27me3 via their chromobox subunits and PRC2 complexes bind to PRC1-catalyzed H2AK119ub1 via their accessory JARID2 and AEBP2 subunits (*40*, *41*). While subunits of the PRC1 and PRC2 complexes are, for the most part, in separate biochemical entities, studies of *Drosophila* Polycomb complexes suggest that PRC1 and PRC2 complexes interact via Scm protein (SCMH1, SCML1, and SCML2 in human) acting as a bridge. Our demonstration that rTetR-CBX7 could establish H3K27me3 in RING1A/RING1B double-knockout cells, together with coimmunoprecipitation of PRC1 and PRC2 subunits (Figs. 5A and 6, B and C), support interactions between Polycomb complexes in the absence of the histone modifications catalyzed by each complex. Furthermore, AF-M predicts interactions across Polycomb complexes, which require further experimental validation. While PRC2/EZH2 efficiently catalyzes H3K27 trimethylation, PRC2/EZH1 has been shown to compact chromatin and repress transcription in vitro (*77*). The noncatalytic function of PRC2 in epigenetic maintenance of silencing may therefore involve its ability to interact with PRC1 complexes and further compact chromatin.

## MATERIALS AND METHODS

### Cell culture

HEK293FT (Thermo Fisher Scientific, R70007) cells were maintained in Dulbecco’s minimum essential medium (Invitrogen) plus 10% fetal bovine serum (Invitrogen), 1 mM glutamine, and penicillin-streptomycin (100 μg/ml) following standard culture conditions. To induce the binding rTetR-CBX7 to the *5xtetO* Site, doxycycline (1 μg/ml; Sigma-Aldrich, D9891) was added to the culture medium. The reagents used in this study are listed in table S1. The cell lines generated in this study are listed in tables S2 and S3.

### Plasmid construction

Donor plasmids for insertion of *5xtetO-pEF1-H2B-CITRINE* reporter into the genome were constructed by subcloning *5xtetO-pEF1-H2B-CITRINE-PolyA* from PhiC31-Neo-ins-5xtetO-pEF-H2B-Citrine-ins (Addgene, no. 78099) (*15*) with right and left homology arms (500 bps each) in CloneSmart HCKan Blunt [from J. Zhang (Initiative for Genome Editing and Neurodegeneration core in the Department of Cell Biology at Harvard Medical School), Lucigen, no. 40704-2). Plasmid with mCherry-2A-rTetR-CBX7 was created by subcloning mCherry-2A-rTetR (Addgene, no. 78101) (*15*) and CBX7 into lentiviral expression vector backbone pLVU-tTR-KRAB (Addgene, no. 11645) (*78*). The CBX7 open reading frame was amplified from pCMV-SPORT6-CBX7 (DFCI, plasmid no. HsCD00339744). A 3xFlag epitope was inserted at the C terminus of CBX7 to generate mCherry-2A-rTetR-CBX7-3xFlag. Rescue plasmids were constructed by cloning HA or 3X Flag tags fused to EED, MTF2, and RYBP wild-type and mutant cDNAs using Gibson assembly into pdCas9-DNMT3A-2A-PuroR (Addgene, no. 71667) (*79*), replacing pdCas9-DNMT3A. In the case of RYBP, PuroR was replaced with hygromycin. The point mutations in EED, MTF2, and RYBP were generated using IDT gBlocks.

### CRISPR genome editing

Single guide RNAs (sgRNAs) for reporter cell line construction, gene knockouts, and sequence deletions were designed using the CRISPR design tool in https://benchling.com and/or https://chopchop.cbu.uib.no/ (table S2). sgRNAs were either in vitro transcribed using GeneArt Precision gRNA Synthesis Kit (Thermo Fisher Scientific, A29377) and electroporated with Neon Transfection System (Thermo Fisher Scientific, MPK1025), along with donor plasmid and Cas9 protein (from J. Zhang, Initiative for Genome Editing and Neurodegeneration core in the Department of Cell Biology at Harvard Medical School) or cloned into pSpCas9 (BB)-2A-Puro (PX459) V2.0 (Addgene, plasmid no. 62988) and transfected into HEK293FT using Lipofectamine 2000 (Thermo Fisher Scientific,). sgRNAs for rTetR-CBX7 deletion were cloned into LentiCRISPRv2 (Addgene, 52961). CITRINE-positive cells were sorted into single-cell colonies in 96-well plates, genotyped by PCR (genotyping primer sequences are presented in table S4) and confirmed by Sanger sequencing (Quintara Bio) or MiSeq (Illumina). Southern blot using a CITRINE probe was carried out to verify single integration for the reporter cell lines.

### Integration of rescue constructs

Rescue plasmids were transfected into relevant cell lines using Lipofectamine 2000 (Thermo Fisher Scientific). Cells with insertions were selected using puromycin (Thermo Fisher Scientific) at 0.6 μg/ml or hygromycin (Thermo Fisher Scientific) at 200 μg/ml for 2 weeks. See table S3.

### Western blot

Whole-cell extract was obtained by lysis in RIPA buffer [final concentrations: 150 mM NaCl, 1% Triton X-100, 0.5% sodium deoxycholate, 0.1% SDS, 50 mM tris (pH 8.0)] and histones were extracted using 0.2 N HCl. The protein concentration was determined by the Bradford assay (BioRad). Ten to 20 micrograms per lane of total protein was electrophoresed on 4 to 15% Mini-PROTEAN TGX Precast Protein Gels (BioRad) with SDS running buffer and transferred to polyvinylidene difluoride membranes. The membranes were blocked [5% nonfat dry milk in 1× phosphate-buffered saline (PBS), 0.1% Tween 20] for 2 hours and then incubated in 5% nonfat dry milk in 1× PBS (137 mM NaCl, 2.7 mM KCl, 8 mM Na_2_HPO_4_, and 2 mM KH_2_PO_4_.), 0.1% Tween 20 with the primary antibodies as listed in table S5 for 2 hours at room temperature or overnight at 4°C. Last, membranes were incubated with corresponding secondary Licor IRDye antibody (5% nonfat dry milk in 1× PBS, and 0.1% Tween 20) and imaged by Odyssey Clx (Licor) or horseradish peroxidase–conjugated secondary antibodies and imaged on autoradiography film/Amersham Imager (GE).

### RT-qPCR

Total RNA was extracted using the rNeasy Mini kit (74104, Qiagen) and reverse transcribed into cDNA using random hexamers (Invitrogen) and reverse transcription kit (18090010, Thermo Fisher Scientific). cDNA was analyzed using PCR on a QuantStudio 7 Flex Real Time PCR System (Applied Biosystem). PCR parameters were 95°C for 2 min and 40 cycles of 95°C for 15 s, 60°C for 30 s, and 72°C for 15 s, followed by 72°C for 1 min. All the qPCR data presented were at least two biological replicates and plotted with Prism GraphPad Software with error bars representing SD. Primer sequences are presented in table S6.

### Lentiviral production and infection

Plasmids were purified using a MaxiPrep DNA isolation Kit (Qiagen). For virus packaging, we used psPAX2 (Addgene, no. 12260) and pMD2.G (Addgene, no. 12259), which were transfected into HEK293FT cells using Lipofectamine 2000 (Invitrogen). Medium containing the viral particles was collected 72 hours after transfection and viral particles were concentrated using the PEG-it Virus precipitation solution (SBI LV810A-1). Cells were transduced with the virus for 48 hours in the presence of polybrene (4 μg/ml; Sigma-Aldrich, H9268).

### Fluorescence imaging

Cells were plated on chamber slides (Thermo Fisher Scientific, 154526PK). Cells were first washed with PBS, fixed with 4% paraformaldehyde in PBS for 5 min and permeabilized with PBS/0.25% Triton X-100 at room temperature for 5 min. Cells were mounted with VECTASHIELD HardSet Antifade Mounting Medium with 4′,6-diamidino-2-phenylindole (DAPI; Vector Labs) and imaged in the DAPI, yellow fluorescent protein, and red fluorescent protein channels using a wide-field microscope (Nikon Ti2) equipped with a 40× objective lens (Nikon Imaging Center at Harvard Medical School). Images were postprocessed with ImageJ (*80*).

### Fluorescence-activated cell sorting and analysis

Cells were made into single cell suspension using 0.05% trypsin (Invitrogen) and suspended in HEK293FT culture medium. Samples for analysis were collected with LSR Fortessa (BD Biosciences, Dana Farber Flow Cytometry core) or FACS Calibur (BD Biosciences, Department of Cell Biology at Harvard Medical School). The green fluorescent protein channel was used for CITRINE detection. Samples were sorted with M AriaII (BD Biosciences, Immunology Flow Cytometry core at Harvard Medical School). Data were analyzed with FlowJo Version 10.5.3 (Ashland, OR: Becton, Dickinson and Company; 2021). Experiments were performed with at least two biological replicates.

### ChIP-qPCR and ChIP-seq

ChIP was performed as previously described with minor modifications. Cells for ChIP were cultured in 15-cm plates (~10 million cells). Cell pellets were first washed with cold PBS, cross-linked at room temperature with 1% formaldehyde (Thermo Fisher Scientific) for 8 min. Cross-linking reactions were quenched by addition of 125 mM glycine for 10 min. Cell were then resuspended in swelling buffer [25 mM Hepes (pH 7.8), 1.5 mM MgCl_2_, 10 mM KCl, 0.1% NP-40, and 1 mM dithiothreitol (DTT)] followed by Dounce homogenization. Nuclei were pelleted by centrifugation and then resuspended in sonication buffer [0.1% SDS, 1 mM EDTA, and 10 mM tris-HCl (pH 8.0)]. The nuclei were sonicated to shear chromatin into ∼200 to 500 bp fragments using a Covaris E220. Sonicated samples were diluted with ChIP dilution buffer [0.1% SDS, 1 mM EDTA and 10 mM tris-HCl (pH 8.0), 1% Triton X-100, and 150 mM NaCl]. Diluted samples were centrifuged at 13,000 rpm for 10 min. The supernatant was used for immunoprecipitation using antibodies and 25-μl protein A/G beads for 12 to 16 hours at 4°C (see table S5 for antibodies). For H3K27me3 ChIP-Seq, *Drosophila* S2 chromatin (Active Motif, no. 53083) and histone H2Av antibody (Active Motif, no. 61686) were added as spike-in controls. ChIP-seq samples for Flag antibody do not have spike-in controls. The beads were washed twice with high-salt wash buffer A [50 mM Hepes (pH 7.9), 500 mM NaCl, 1 mM EDTA, 1% Triton X-100, 0.1% sodium deoxycholate, and 0.1% SDS], twice with wash buffer B [20 mM tris-HCl (pH 8.0), 250 mM LiCl, 1 mM EDTA, 0.5% sodium deoxycholate, and 0.5% NP-40], and twice with 1× TE (10 mM tris-HCl and 1 mM EDTA). The bound chromatin fragments were eluted with elution buffer [50 mM tris (pH 8.0), 1 mM EDTA, 50 mM NaHCO_3_, and 1% SDS] twice for 10 min each at 65°C. Eluted DNA-proteins complexes were incubated overnight at 65°C to reverse cross-links. RNAase A followed by proteinase K was then added to digest RNA and protein. DNA was further purified using phenol chloroform/PCR purification kit (Qiagen) and analyzed by PCR on a QuantStudio 7 Flex Real Time PCR System (Applied Biosystem). PCR parameters were 95°Cfor 2 min and 40 cycles of 95°C for 15 s, 60°C for 30 s, and 72°C for 15 s, followed by 72°C for 1 min. All the ChIP-qPCR data presented include at least two biological replicates. Primer sequences are in table S7. Results were plotted with Prism GraphPad Software with error bars representing SD.

For ChIP-seq, sequencing libraries were constructed using TruSeq DNA sample Prep Kits (Illumina) and adapter dimers were removed by 2% agarose and tris-acetate-EDTA gel electrophoresis. Size-selected and purified DNA libraries were sequenced on an Illumina NextSeq 500 machine (Bauer core facility at Harvard University) to obtain 75-bp single-end reads. ChIP-seq reads were quality controlled with fastqc (v0.11.5) and mapped to the human genome reference (Custom *5xtetO-H2B-CITRINE* reporter inserted at Chr11-hg19 near *WT1* or custom *5xtetO-H2B-CITRINE* reporter inserted at Chr3-hg19 near *TFRC*) and *Drosophila* (dm3) using Bowtie2 (v2.2.9) with default parameters. Scale factor was calculated as previously described to normalize H3K27me3 signal. Bam files were generated with SAMtools 1.3.1, which was followed by making bigwig files with deepTools (v/3.0.2) (*81*, *82*). Reads were normalized with scale factor for H3K27me3 or reads per genome coverage (RPGC) for Flag with deepTools (v/3.0.2) bamCoverage function. ChIP-seq tracks were visualized in Integrative Genomics Viewer. Publicly available source data used for this study are listed in table S8.

### rTetR-CBX7-3xFlag purification and MS analysis

Immunoprecipitation and MS analysis were performed as described previously with modifications (*83*). Cells were washed with ice-cold PBS and then resuspended in ice-cold hypotonic buffer [10 mM Hepes, (pH 7.9), 1.5 mM MgCl_2_, 10 mM KCl, 0.2 mM phenylmethylsulfonyl fluoride (PMSF), and 0.2 mM DTT] for 10 min. Plasma membranes were then disrupted by douncing 10 times. Nuclei were pelleted by centrifugation at 2000*g* for 3 min and then resuspended in IP buffer [20 mM tris-HCl (pH 7.9), 250 mM NaCl, 10% glycerol, 2.5 mM MgCl_2_, 0.5 mM CaCl_2_, 0.1 mM EDTA, and 0.5% Triton X-100] containing protease inhibitor cocktail (5056489001, Sigma-Aldrich) and 1 mM deoxyribonuclease I (DNase I). DNA was digested for 1 hour at 4C and centrifuged at 10,000*g* for 10 min. The supernatant was then incubated with antibodies and immune complexes were collected using Dynabeads Protein G (Thermo Fisher Scientific). MS analysis was performed as described previously (*83*). In brief, the Dynabeads Protein G–binding proteins were eluted with 0.5 M NH_4_OH, then resuspended in 200 mM 4-(2-Hydroxyethyl)-1-piperazinepropanesulfonic acid (EPPS, pH 8.5) and digested with trypsin (5 ng/μl). A Q Exactive mass spectrometer (Thermo Fisher Scientific, San Jose, CA) and a Comet-based in-house software pipeline were sequentially used for collecting MS spectra. MS spectra were then converted to mzXML using a modified version of ReAdW.exe. For statistical analysis, *P* values were generated using one-tailed, two-sample unequal variance Student’s *t* test on spectral count of proteins normalized to their protein length.

### Coimmunoprecipitation

For coimmunoprecipitaion, cells were collected from one 15-cm plate, washed with ice-cold PBS and then resuspended in ice-cold hypotonic buffer [10 mM Hepes (pH 7.9), 1.5 mM MgCl_2_, 10 mM KCl, 0.2 mM PMSF, and 0.2 mM DTT] for 10 min. Plasma membranes were then disrupted by douncing. Nuclei were pelleted by centrifugation at 2000*g* for 3 min and then resuspended in IP buffer [20 mM Hepes (pH 7.4), 150 mM NaCl, 10% glycerol, 1 mM MgCl_2_, 1 mM EGTA, and 0.5% Triton X-100] containing protease inhibitor cocktail (5056489001, Sigma-Aldrich) and 1 mM DNase I. DNA was digested for 2 hours at 4°C followed by centrifuged at 10,000*g* for 10 min. The supernatant was then incubated with specific antibodies and immune complexes were collected using Dynabeads Protein A (Thermo Fisher Scientific). Samples/beads were boiled in SDS loading buffer for 5 min before loaded to SDS-PAGE gel. Immunoblotting was performed as described earlier.

### GST pulldown

GST-BCORL (S1474-G1644) was expressed in *Escherichia coli* strain BL21 by induction with 200 μM isopropyl-β-d-thiogalactopyranoside at 16°C overnight. Bacteria were then collected and washed with cold PBS and sonicated (Branson sonicator) at 4°C in lysis buffer [20 mM Hepes (pH 7.4), 150 mM NaCl, 5% glycerol, 1 mM DTT, and 0.1% Triton X-100] containing protease inhibitor cocktail (5056489001, Sigma-Aldrich). After centrifugation at 10,000*g* for 10 min, the supernatant was added to 0.5-ml Glutathione Sepharose 4B resin (GE Healthcare, 17075605), which was equilibrated with lysis buffer. The resin was then washed six times with wash buffer [20 mM Hepes (pH 7.4), 250 mM NaCl, 5% glycerol, 1 mM DTT, and 0.2% Triton X-100], eluted with 20 mM reduced glutathione, and the elution was dialyzed in dialysis buffer [20 mM Hepes (pH 7.4), 250 mM NaCl, 10% glycerol, and 1 mM DTT] overnight to remove glutathione. Strep-RBBP7 was transiently transfected and expressed in HEK293FT cell for at least 48 hours. Cells were then collected and washed with cold PBS and lysed in lysis buffer [20 mM Hepes (pH 7.4), 150 mM NaCl, 10% glycerol, 1 mM MgCl_2_, 1 mM EGTA, and 1% Triton X-100] containing protease inhibitor cocktail (5056489001, Sigma-Aldrich), DNase I, and benzonase. DNA and RNA were digested for 2 hours at 4°C and centrifuged at 10,000*g* for 10 min. The supernatant was loaded to Strep-Tactin Sepharose resin (IBA, 2-1201-002), washed with lysis buffer, and eluted by 2.5 mM desthiobiotin.

For GST pulldown assays, 10-μl 50% slurry of Glutathione Sepharose 4B was used for each sample. GST-BCORL (S1474-G1644) (0.1 μM) was incubated with Strep-RBBP7 (0.1 μM) in lysis buffer containing 0.1% Triton X-100 overnight at 4°C. Beads were washed four times with lysis buffer containing 0.1% Triton X-100, resuspended in SDS sample buffer, and boiled for 5 min. Input (2 to 5%) and bound proteins (10 to 50%) were run on 4 to 20% TGX stain-free SDS-PAGE gel (Bio-Rad, no. 4568096) and analyzed by Bio-Rad Chemidoc MP imaging system.

### AF-M structural predictions

Structural predictions in this study were performed with template-free mode of AlphaFold2-Multimer v3 with recycling number 5 using localColabFold at Harvard Medical School local computational cluster O_2_. Amino acid sequences used for structural predictions were obtained from UniProtKB database. Five possible structural models were provided by each prediction. Predicted local distance difference test (pLDDT), pTM, and ipTM score of all five models were collected. Evaluation of the predicted structures were carried out first by plotting the first rank ipTM score heatmap and average ipTM score to visualize the confidence of the predicted protein-protein interactions. High confident predicted structures were then analyzed in UCSF Chimera X-1.6.1 to identify the predicted interaction interfaces. Structural predictions of the entire core PRC2, cPRC1.4, and vPRC1.1 complexes were performed using recycling number 20 to validate the quality of the pairwise predictions. To evaluate the predicted structures, published x-ray crystal or cryo–electron microscopy (cyro-EM) structures, including PRC2-AEBP2-JARID2 bound to H2AK119Ub1 nucleosome cryo-EM structure [Protein Data Bank (PDB): 6WKR] (*40*), RING1B-PCGF4-UBC5HC1 PRC1 ubiquitination module bound to nucleosome core particle crystal structure (PDB: 4R8P) (*84*), and other structures noted in the text or figure legends were used to analyze the quality of the predicted structures. All pairwise predicted structures and customized structural predictions are included in table S11.
