# Supplementary material for: Genomic context– and H2AK119 ubiquitination–dependent inheritance of human Polycomb silencing
Source: Sci Adv. 2024 May 8;10(19):eadl4529. doi: 10.1126/sciadv.adl4529 (PMC11078181; doi:10.1126/sciadv.adl4529)
Supplement: Supplementary file 1 — Figs. S1 to S11 Tables S1 to S9 Legends for tables S10 and S11 [file sciadv.adl4529_sm.pdf]

Science Advances
AAAS

Supplementary Materials for

Genomic context– and H2AK119 ubiquitination–dependent inheritance of human Polycomb silencing

Tiasha A. Shafiq *et al.*

Corresponding author: Danesh Moazed, danesh@hms.harvard.edu

Sci. Adv. **10**, eadl4529 (2024)
DOI: 10.1126/sciadv.adl4529

The PDF file includes:

Figs. S1 to S11
Tables S1 to S9
Legends for tables S10 and S11

Other Supplementary Material for this manuscript includes the following:

Tables S10 and S11

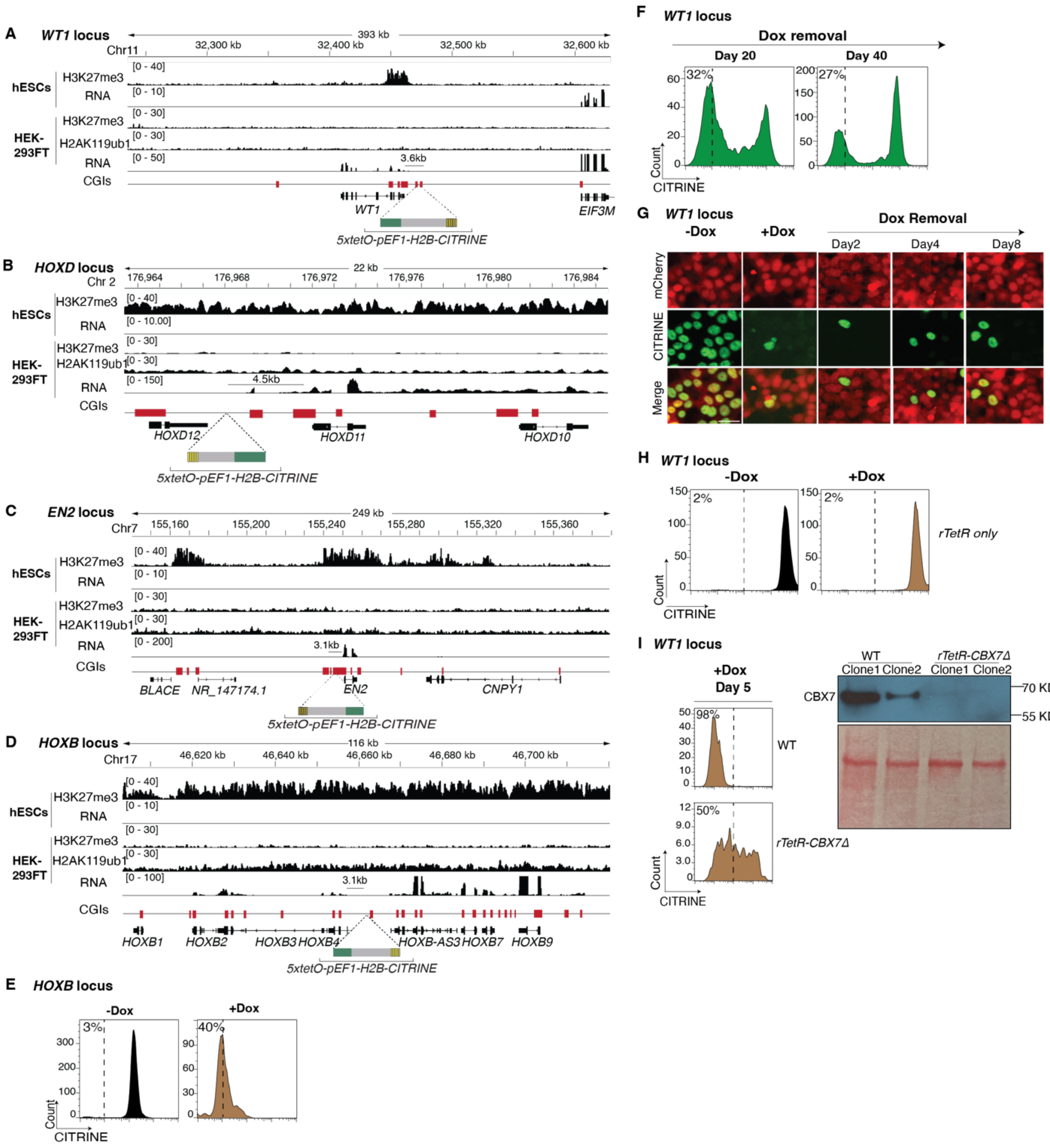

Fig. S1. Chromatin features and additional control experiments at reporters inserted near developmental genes. Genome browser tracks showing publicly available chromatin features of the *WT1* (**A**), *HOXD* (**B**), *EN2* (**C**) and *HOXB* (**D**) loci in human embryonic stem cells (hESCs) and HEK293FT cells (see Table S8 for references). Chromosome coordinates (top) and the site of insertion of the *5xtetO-pEF1-H2B-CITRINE* reporter (bottom) are indicated. **E,** Flow

cytometry histograms showing CITRINE expression before and after establishment of silencing (-Dox and +Dox) near *HOXB4*. Percentages (%) indicate the fraction of CITRINE negative cells. **F,** Flow cytometry histograms showing CITRINE expression at the *WT1* locus at 20 and 40 days after removal of doxycycline (Dox removal). Percentages (%) indicate the fraction of CITRINE negative cells. **G,** Representative fluorescence images showing CITRINE expression at the *WT1* locus before and after establishment of silencing (-Dox and +Dox) and at the indicated days after removal of doxycycline (Dox removal). mCherry indicates the expression of rTetR-CBX7 in the reporter cell line. Scale bar, 25 μM. **H,** Flow cytometry histogram showing CITRINE expression at the *WT1* locus before and after doxycycline addition (-Dox and +Dox) in a cell line expressing rTetR-only. Percentages (%) indicate the fraction of CITRINE negative cells. **I,** Flow cytometry histograms showing CITRINE expression at the *WT1* locus in cells with rTetR-CBX7 excised after establishment with doxycycline addition (+Dox) and western blot showing the expression levels of rTetR-CBX7 before and after excision. Clone 1 and Clone 2 are independent clonal lines carrying rTetR-CBX7 and *5xtetO::CITRINE* inserted at the *WT1* locus in which we deleted rTetR-CBX7 using CRISPR/Cas9. Clone 2 with rTetR-CBX7 was used for FACS analysis.

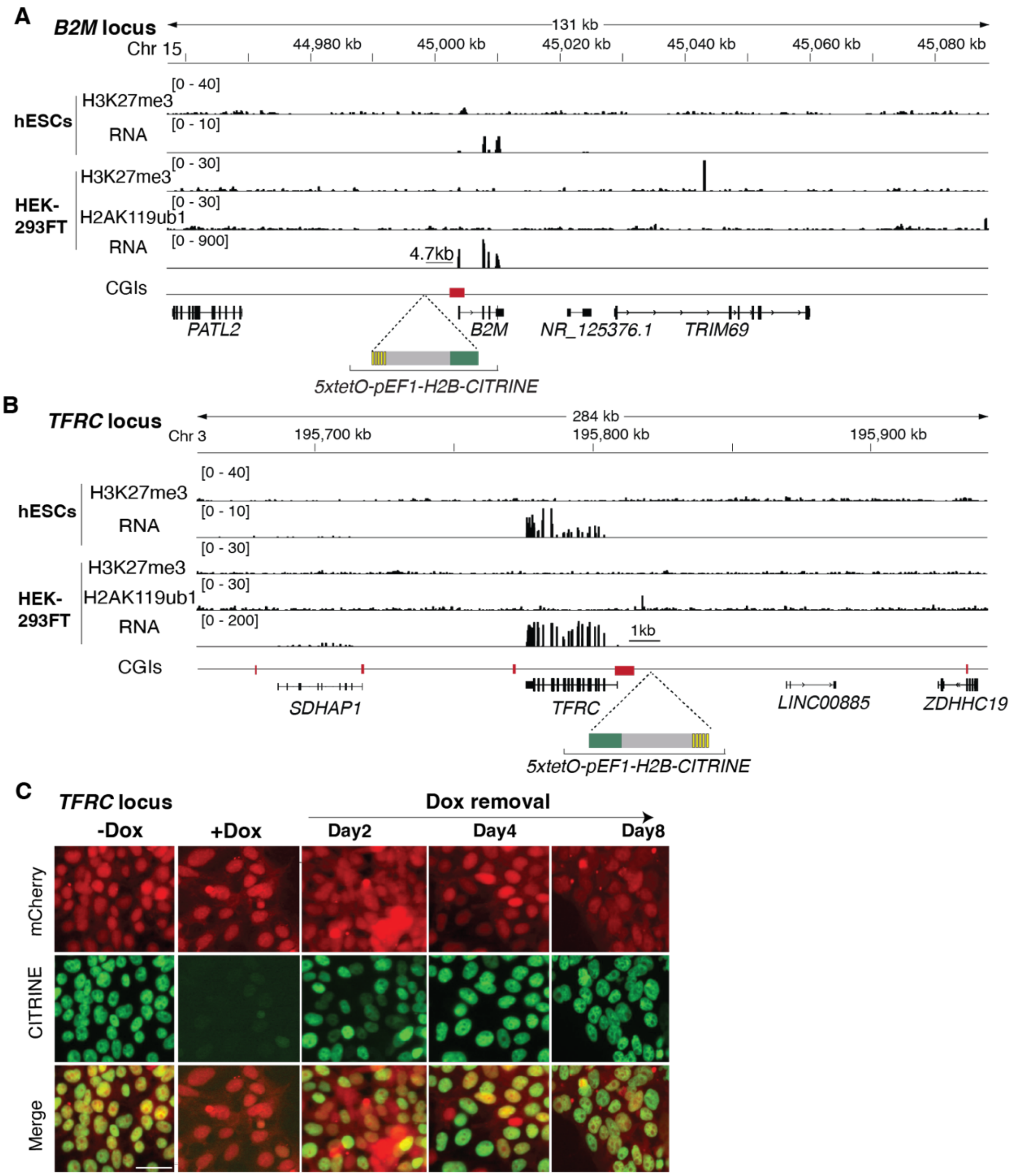

Fig. S2. Chromatin features and additional control experiments at reporters inserted near ubiquitously expressed genes. Genome browser tracks showing publicly available chromatin features of the *B2M* (**A**) and TFRC (**B**) loci in human embryonic stem cells (hESCs) and HEK293FT cells (see Table S8 for references). Chromosome coordinates (top) and the site of insertion of the *5xtetO-pEF1-H2B-CITRINE* reporter (bottom) are indicated. **C,** Representative fluorescence images showing CITRINE expression before and after establishment of silencing (-

Dox and +Dox) and at the indicated days after removal of doxycycline (Dox removal) at the reporter locus inserted near *TFRC*. mCherry indicates the expression of rTetR-CBX7 in the reporter cell line. Scale bar, 25 μM.

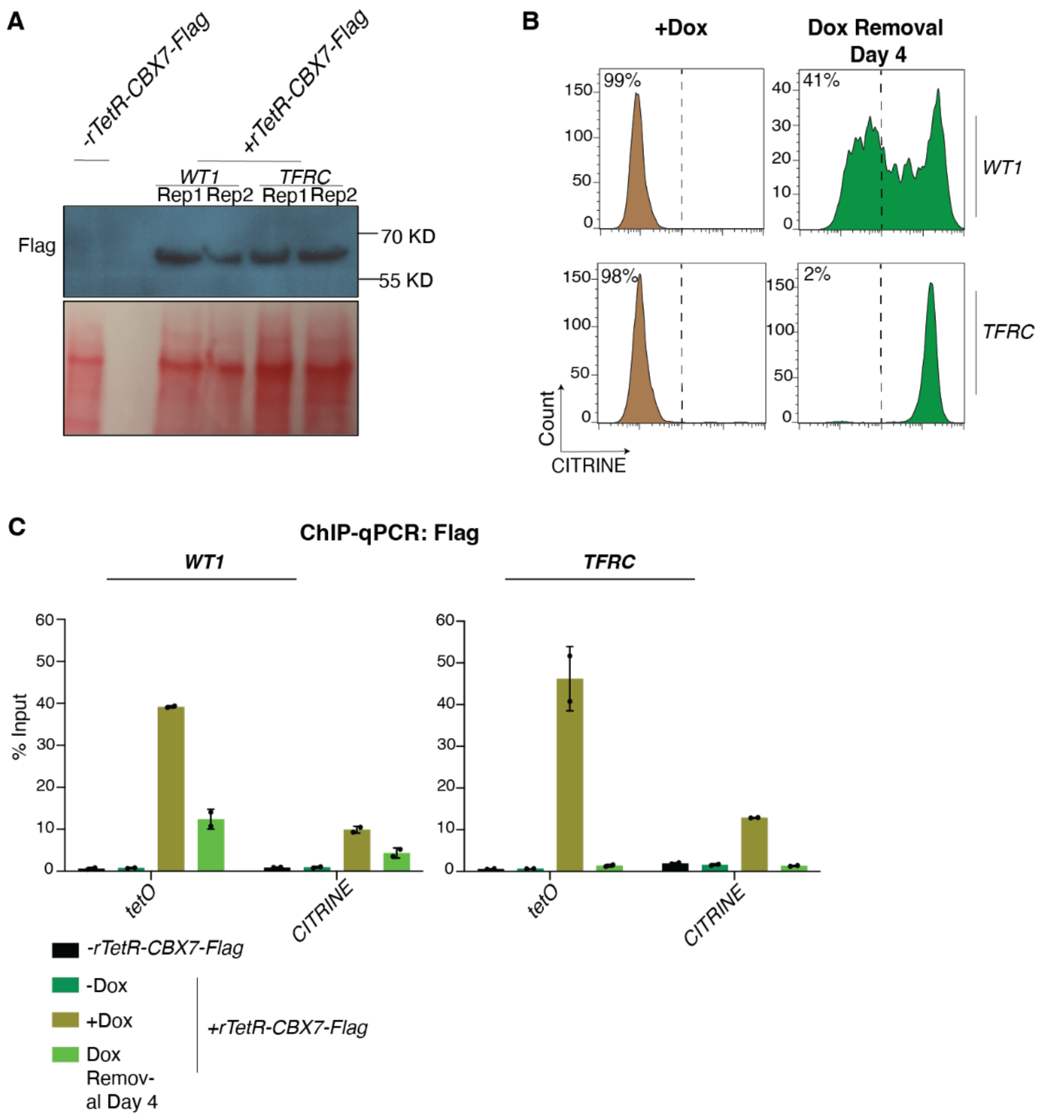

Fig. S3. **Binding and release of rTetR-CBX7-3xFlag to the *tetO* arrays at the *WT1* and *TFRC* loci. A,** Western Blot showing the expression levels of rTetR-CBX7-Flag expressed in the *WT1* and *TFRC* reporter cell lines (top) and Ponceau staining (bottom). **B**, Flow cytometry histograms showing CITRINE expression after establishment of silencing with doxycycline addition (+Dox) and at 4 days after removal of doxycycline (Dox removal) (bottom) in the cell lines in **A**. Percentages (%) indicate the fraction of CITRINE negative cells. **C,** ChIP-qPCR

analysis of Flag at the reporter loci at *WT1* and *TFRC* before establishment (-Dox), after establishment (+Dox), and at 4 days after removal of doxycycline (Dox removal). Reporter cell lines without expression of rTetR-CBX7-Flag served as controls. Error bars represent standard deviations.

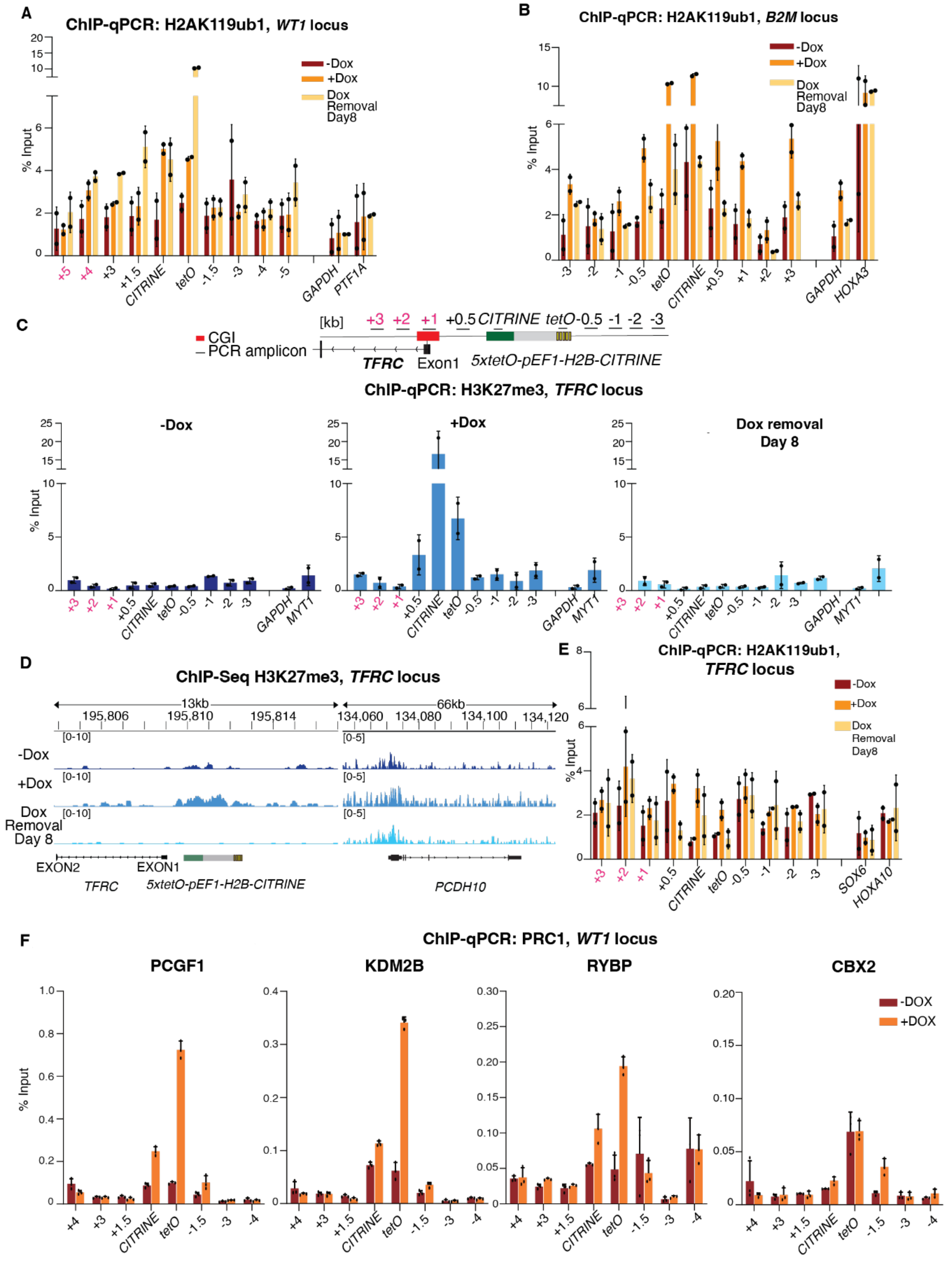

Fig. S4. H2AK119ub1, and H3K27me3 are established at *WT1, B2M* and *TFRC* loci, but only inherited at the *WT1* locus. A, ChIP-qPCR analysis of H2AK119ub1 enrichment at the

reporter locus and surrounding sequences before establishment (-Dox), after establishment (+Dox), and at 8 days after removal of doxycycline (Dox removal) near the *WT1* locus. *GAPDH* served as a negative control and *PTF1A* as a positive control. Error bars represent standard deviations. See Fig. 2 for primer maps. **B**, Same as **A** but at the *B2M* locus. See Fig. 2 for primer maps. **C,** ChIP-qPCR analysis of H3K27me3 enrichment at the reporter locus and surrounding sequences before establishment (-Dox), after establishment (+Dox), and at 8 days after removal of doxycycline (Dox removal) near the *TFRC* locus. *GAPDH* served as a negative control and *MYT1* as a positive control. Error bars represent standard deviations. **D**, Genome browser snapshots of H3K27me3 ChIP-seq reads at the reporter locus near *TFRC* and the endogenous Polycomb-silenced *PCDH10* gene. **E,** Same as **C**, but showing H2AK119ub1 ChIP-qPCR.
F, Same as **A**, but showing ChIP-qPCR for vPRC1 subunits PCGF1, KDM2B, and RYBP, and cPRC1 subunit CBX2.

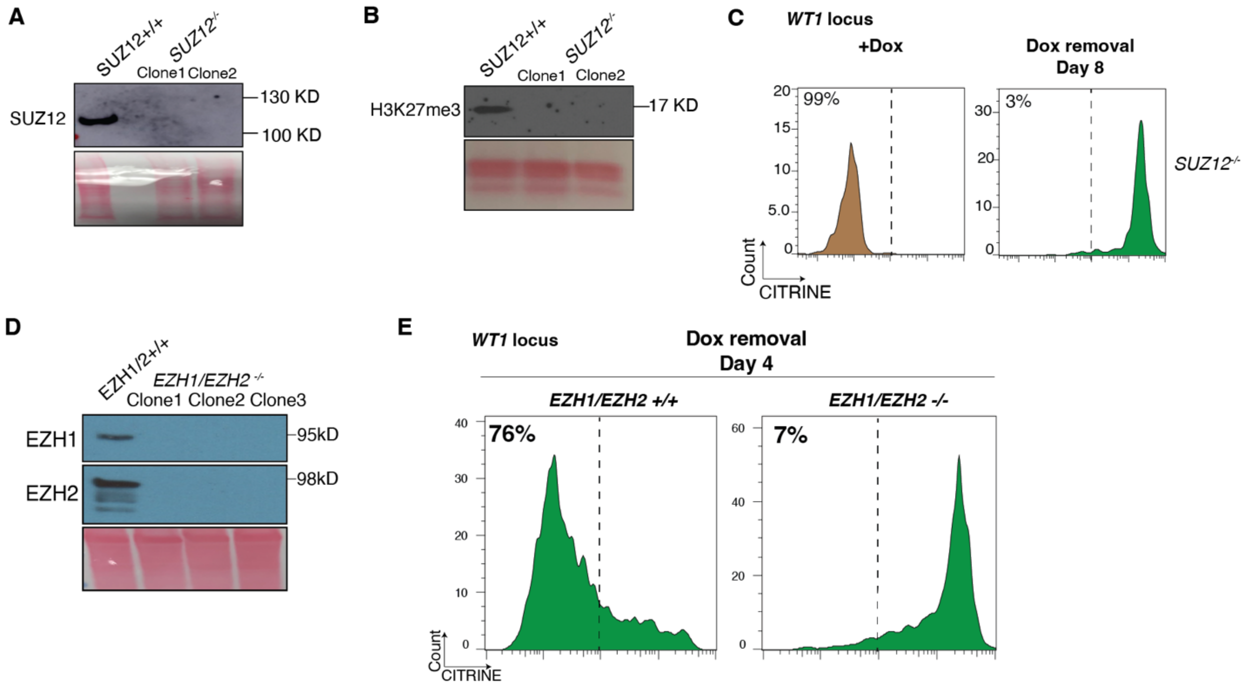

Fig. S5. PRC2 subunits *SUZ12* and EZH1/EZH2 are required for epigenetic inheritance of Polycomb silencing at the *WT1* developmental gene. A, B, Western Blot showing the levels of SUZ12 protein (A) and H3K27me3 (B) in *SUZ12* knockout cell lines (*SUZ12*$^{-/-}$) and wild-type cells (WT). **C,** Flow cytometry histograms showing CITRINE expression after establishment of silencing (+Dox) and 8 days after removal of doxycycline (Dox removal) in *SUZ12*$^{-/-}$ cells. **D,** Western Blot showing the levels of EZH1 and EZH2 protein in *EZH1/EZH2* double knockout cell lines (*EZH1/EZH2*$^{-/-}$) and wild-type cells (WT). **E,** Flow cytometry histograms showing CITRINE expression 4 days after removal of doxycycline (Dox removal) in *EZH1/EZH2*$^{+/+}$ and *EZH1/EZH2*$^{-/-}$ cells.

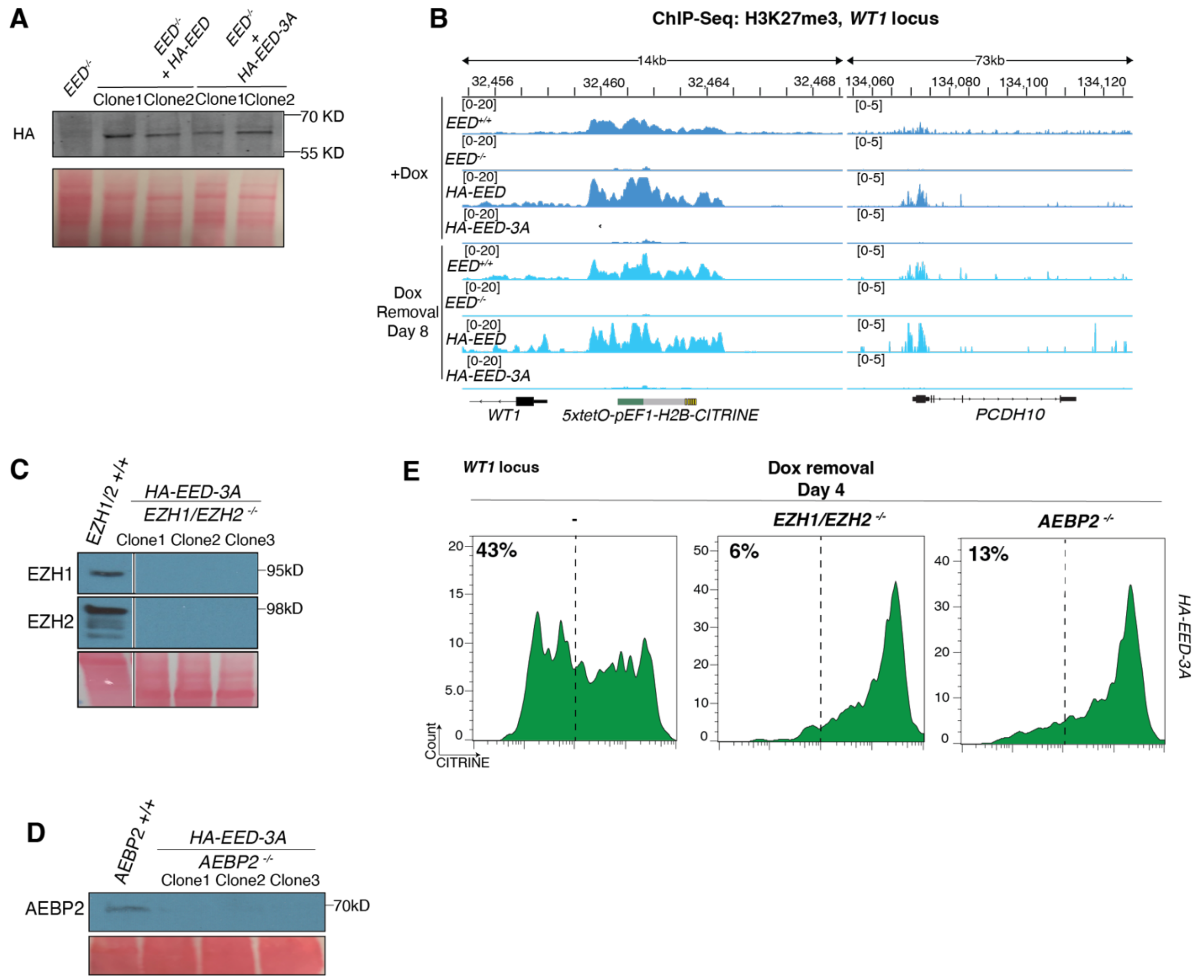

Fig. S6. Aromatic cage mutant *EED* leads to defects in H3K27me3 catalysis, but the mutant EED can be recruited to chromatin. A, Western blot showing the expression levels of HA-EED and HA-EED-3A overexpressed in *EED*$^{-/-}$ cells. **B,** Genome browser snapshots of H3K27me3 ChIP-seq reads at the reporter locus and the endogenous Polycomb-silenced *PCDH10* gene in *EED*$^{+/+}$, *EED*$^{-/-}$, *HA-EED* and *HA-EED-3A* cells after establishment of silencing (+Dox) and 8 days after removal of doxycycline (Dox removal). **C,** Western blot showing the expression levels of EZH2 in wild type (+/+) and *EZH1/EZH2*$^{-/-}$ in cells expressing *HA-EED-3A*. **D**, Western blot showing the expression of AEBP2 in wild type (+/+) and *AEBP2*$^{-/-}$

in cells expressing *HA-EED-3A* cells. **E,** Flow cytometry histograms showing CITRINE expression 4 days after removal of doxycycline (Dox removal) in wild type, *EZH1/EZH2*$^{-/-}$, and *AEBP2*$^{-/-}$ cells.

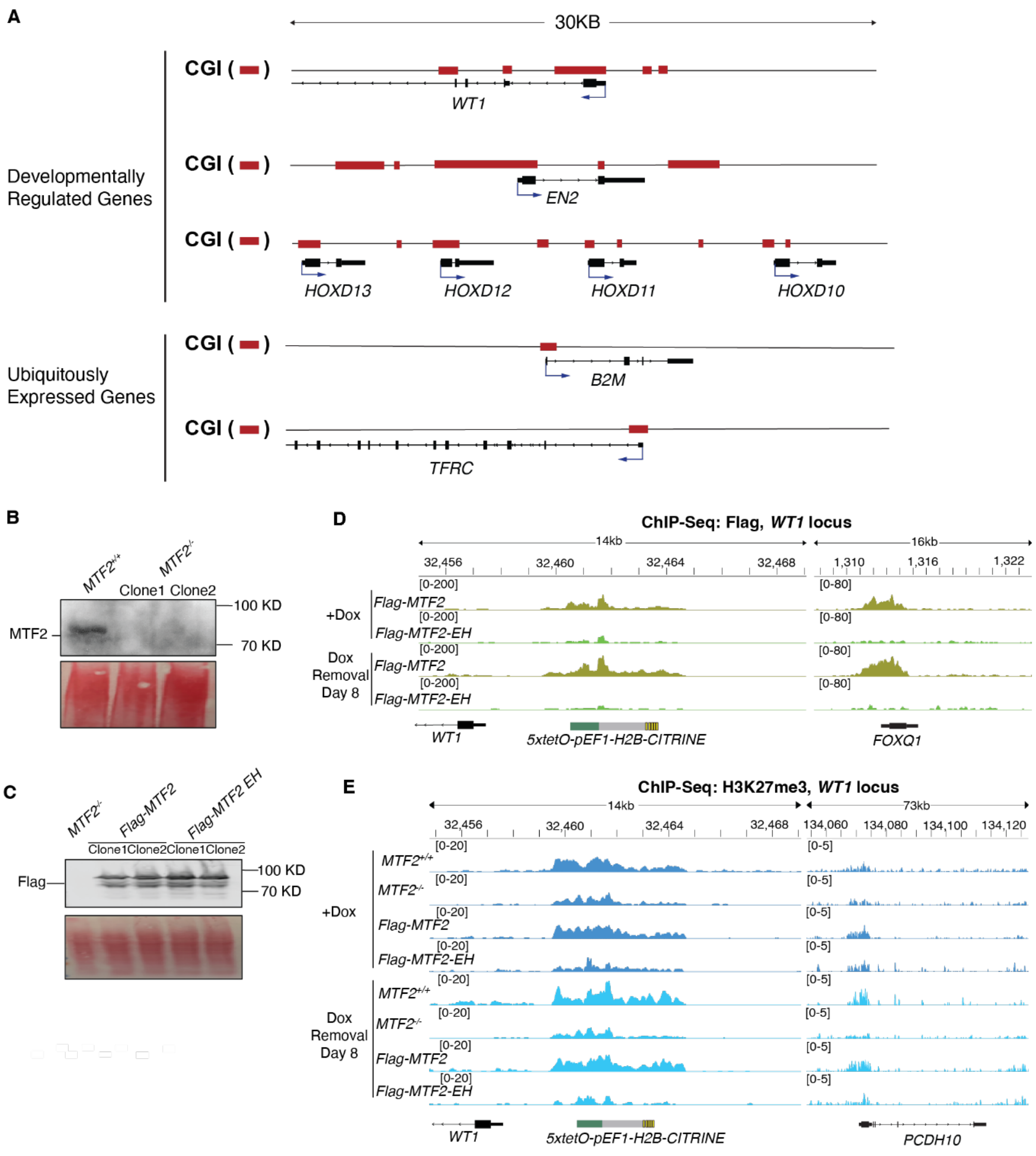

Fig. S7. CpG island density at developmental and housekeeping genes and the effects of mutations in the MTF2 Extended homology domain. A, Genome browser gene tracks highlighting the location of annotated CpG islands (CGIs) at the indicated genes. **B,** Western Blot showing the levels of MTF2 protein in *MTF2* deleted cell line (*MTF2*$^{-/-}$) with the reporter

inserted at the Polycomb target locus*, WT1*. **C,** Western Blot showing the expression levels of *Flag-MTF2* and *Flag-MTF2-EH* overexpressed in *MTF2*$^{-/-}$ cells. **D,** Genome browser snapshots of Flag ChIP-seq reads at the reporter locus and the endogenous Polycomb-silenced *FOXQ1* gene in *Flag-MTF2* and *Flag-MTF2-EH* cells after establishment of silencing (+Dox) and 8 days after removal of doxycycline (Dox removal). **E,** Genome browser snapshots of H3K27me3 ChIP-seq reads at the reporter locus and the endogenous Polycomb-silenced *PCDH10* gene in wild-type (WT, *MTF2*$^{+/+}$), *MTF2*$^{-/-}$ *, Flag-MTF2* and *Flag-MTF2-EH* cells after establishment of silencing (+Dox) and 8 days after removal of doxycycline (Dox removal).

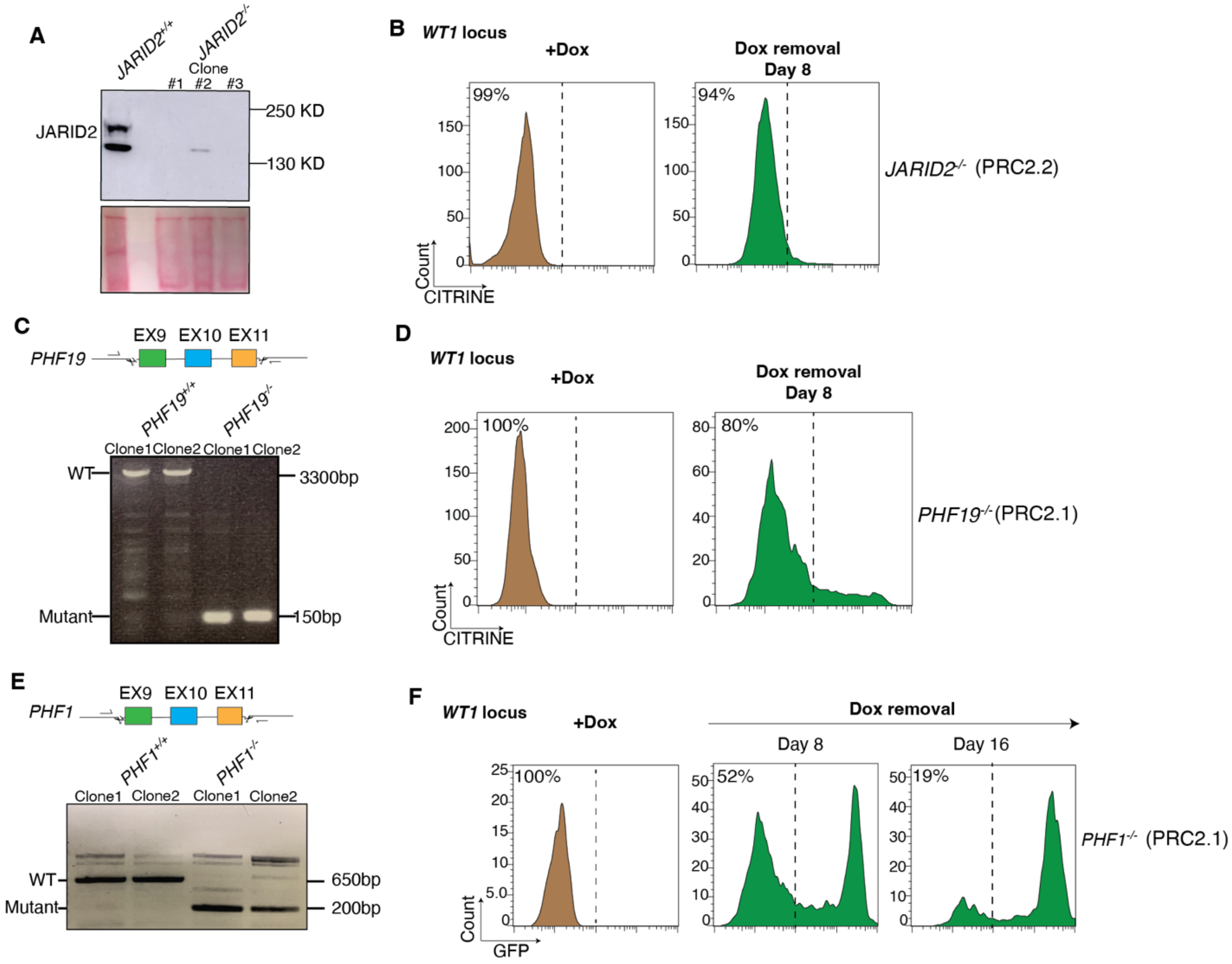

Fig. S8. *PHF1* but not *JARID2* or *PHF19*, contribute to epigenetic inheritance of Polycomb silencing. A, Western Blot showing the levels of JARID2 protein in *JARID2* deleted cell line (*JARID2*$^{-/-}$) and wild-type cells (left). Flow cytometry histograms showing CITRINE expression after establishment of silencing (+Dox) and 8 days after removal of doxycycline (Dox removal) in *JARID2*$^{-/-}$ cells (right). **B,** PCR Genotyping blots validating deletion of exon 9-11 of *PHF19* (left). Flow cytometry histograms showing CITRINE expression after establishment of silencing (+Dox) and 8 days after removal of doxycycline (Dox removal) in *PHF19*$^{-/-}$ cells (right). **C,** PCR genotyping blots validating deletion of exon 9-11 of *PHF1* (left). Flow cytometry histograms showing CITRINE expression after establishment of silencing (+Dox) and 8 and 16 days after removal of doxycycline (Dox removal) in *PHF1*$^{-/-}$ cells (right).

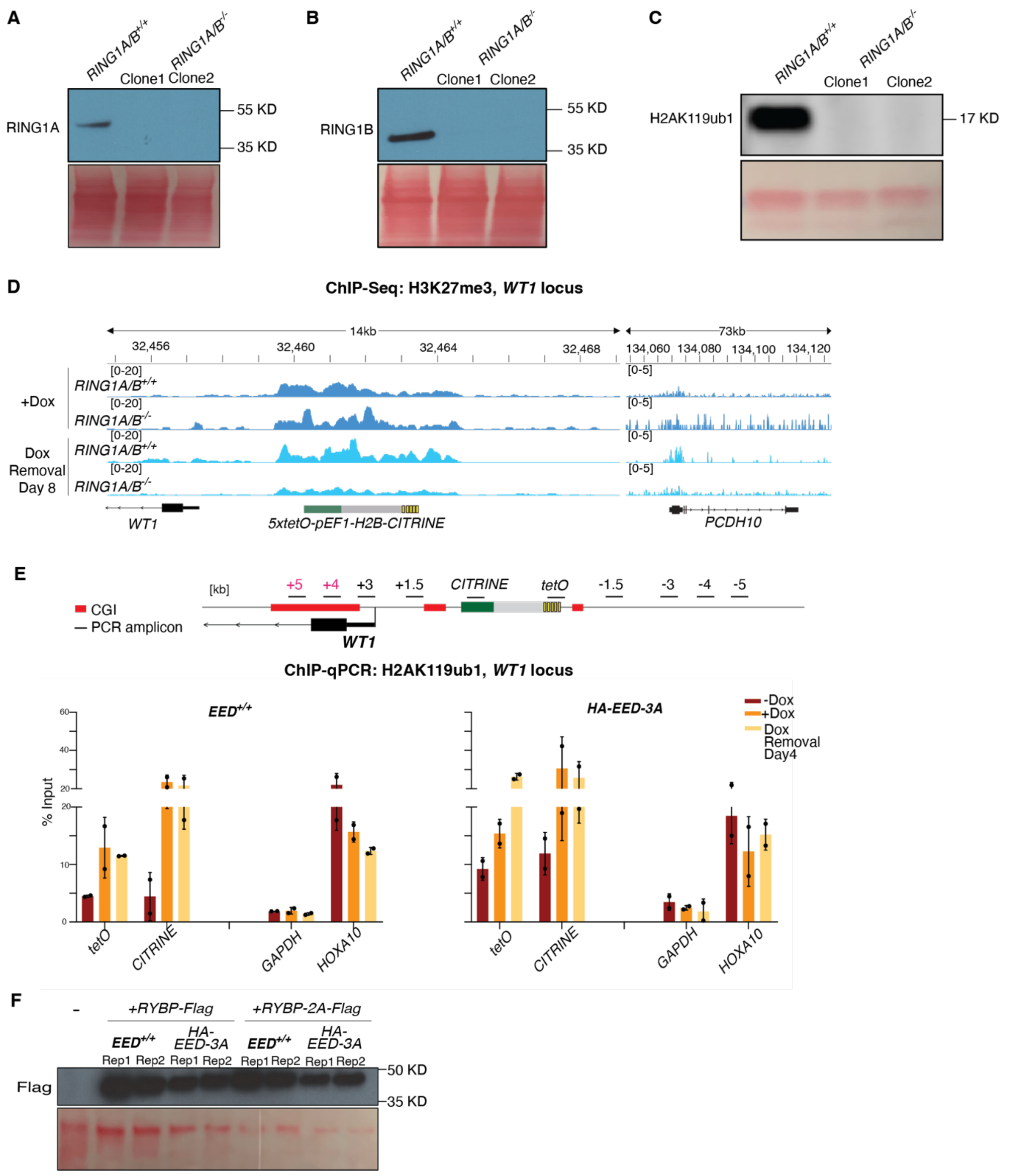

Fig. S9. H2AK119ub1 memory depends on RING1A/B and EED but not the ability of EED to bind to H3K27me3. A, Western Blot showing the expression levels of RING1A protein (top) and RING1B protein (bottom) in wild-type and *RING1A/B*$^{-/-}$ cells. **B,** Western Blot showing the

expression levels of H2AK119ub1 in *RING1A/B*$^{-/-}$ cells. **C,** Genome browser snapshots of H3K27me3 ChIP-seq reads at the *WT1* reporter locus and the endogenous Polycomb-silenced *PCDH10* gene in Control *RING1A/B*$^{+/+}$ and *RING1A/B*$^{-/-}$ cells after establishment of silencing (+Dox) and 8 days after removal of doxycycline (Dox removal). See Fig. 5 for *CITRINE* reporter expression data. **D,** ChIP-qPCR analysis of H2AK119ub1 enrichment at the *CITRINE* reporter, inserted near *WT1*, and surrounding sequences in *EED*$^{+/+}$, and *HA-EED-3A* cells before establishment (-Dox), after establishment (+Dox), and 8 days after removal of doxycycline (Dox removal). *GAPDH* served as a negative control and the native Polycomb-repressed *HOXA10* gene as a positive control. Error bars represent standard deviation. **E,** Western Blot showing the expression levels of wild-type RYBP-Flag and RYBP-2A-Flag proteins in *EED*$^{+/+}$ and *HA-EED-3A* cells. (-) , non-transfected control cells.

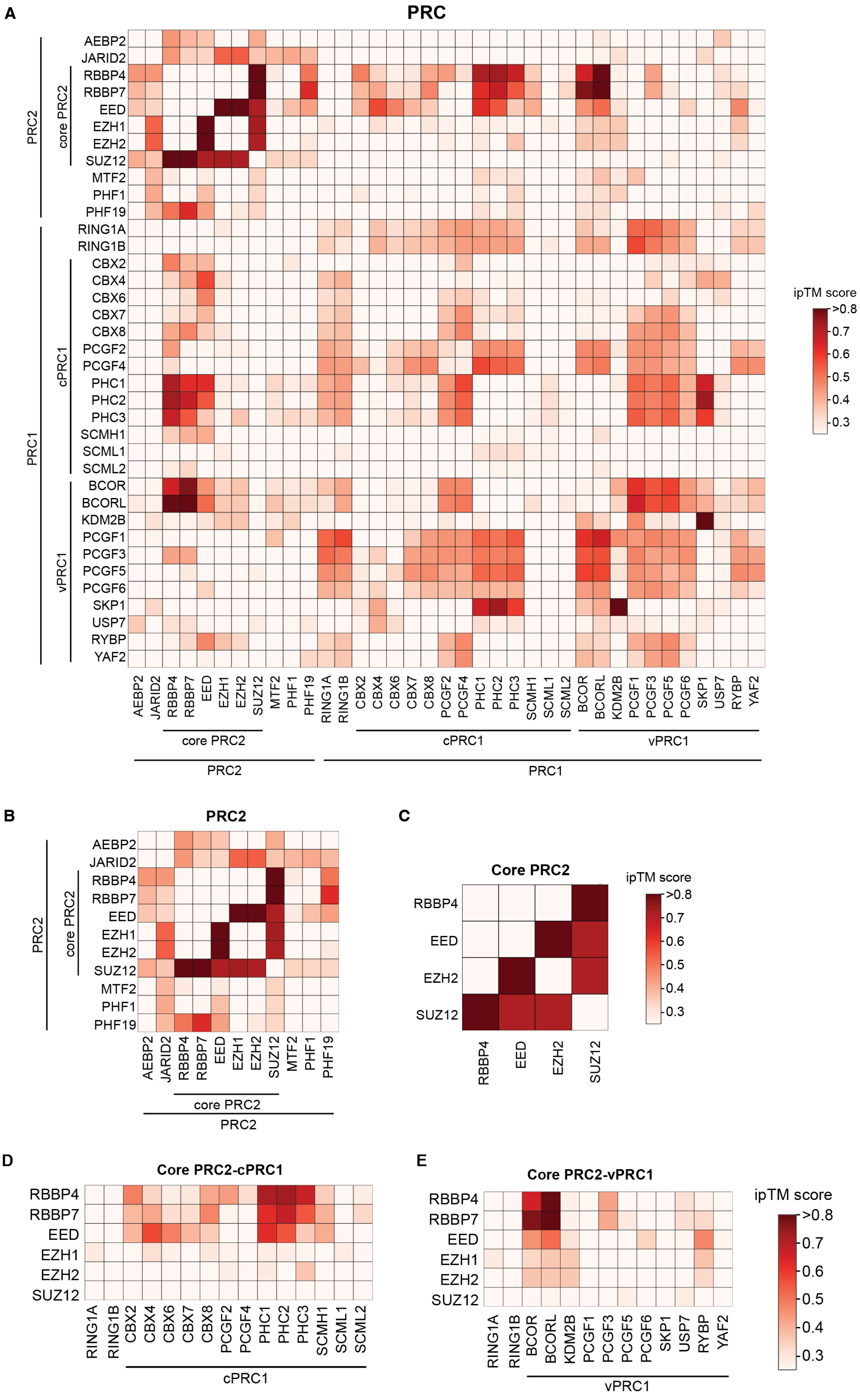
A
PRC
PRC2
core PRC2
PRC1
cPRC1
vPRC1
AEBP2
JARID2
RBBP4
RBBP7
EED
EZH1
EZH2
SUZ12
MTF2
PHF1
PHF19
RING1A
RING1B
CBX2
CBX4
CBX6
CBX7
CBX8
PCGF2
PCGF4
PHC1
PHC2
PHC3
SCMH1
SCML1
SCML2
BCOR
BCORL
KDM2B
PCGF1
PCGF3
PCGF5
PCGF6
SKP1
USP7
RYBP
YAF2
ipTM score
>0.8
0.7
0.6
0.5
0.4
0.3
B
PRC2
C
Core PRC2
D
Core PRC2-cPRC1
E
Core PRC2-vPRC1

Fig. S10. AlphaFold Multimer screen for interactions between subunits of Polycomb complexes. A-C, Heatmaps of AlphaFold-Multimer predictions showing the average interface predicted template modeling (ipTM) score of all five predicted models between the core subunits of the PRC2 and PRC1 complexes (**A**), the subunits of PRC2 and its accessory factors (**B**), and core PRC2 subunits (**C**). **D-E,** Heatmaps of AlphaFold-Multimer predictions showing the average interface predicted template modeling (ipTM) score of all five predicted models between the core subunits of the PRC2 with cPRC1 (**D**) or vPRC1 (**E**) complexes.

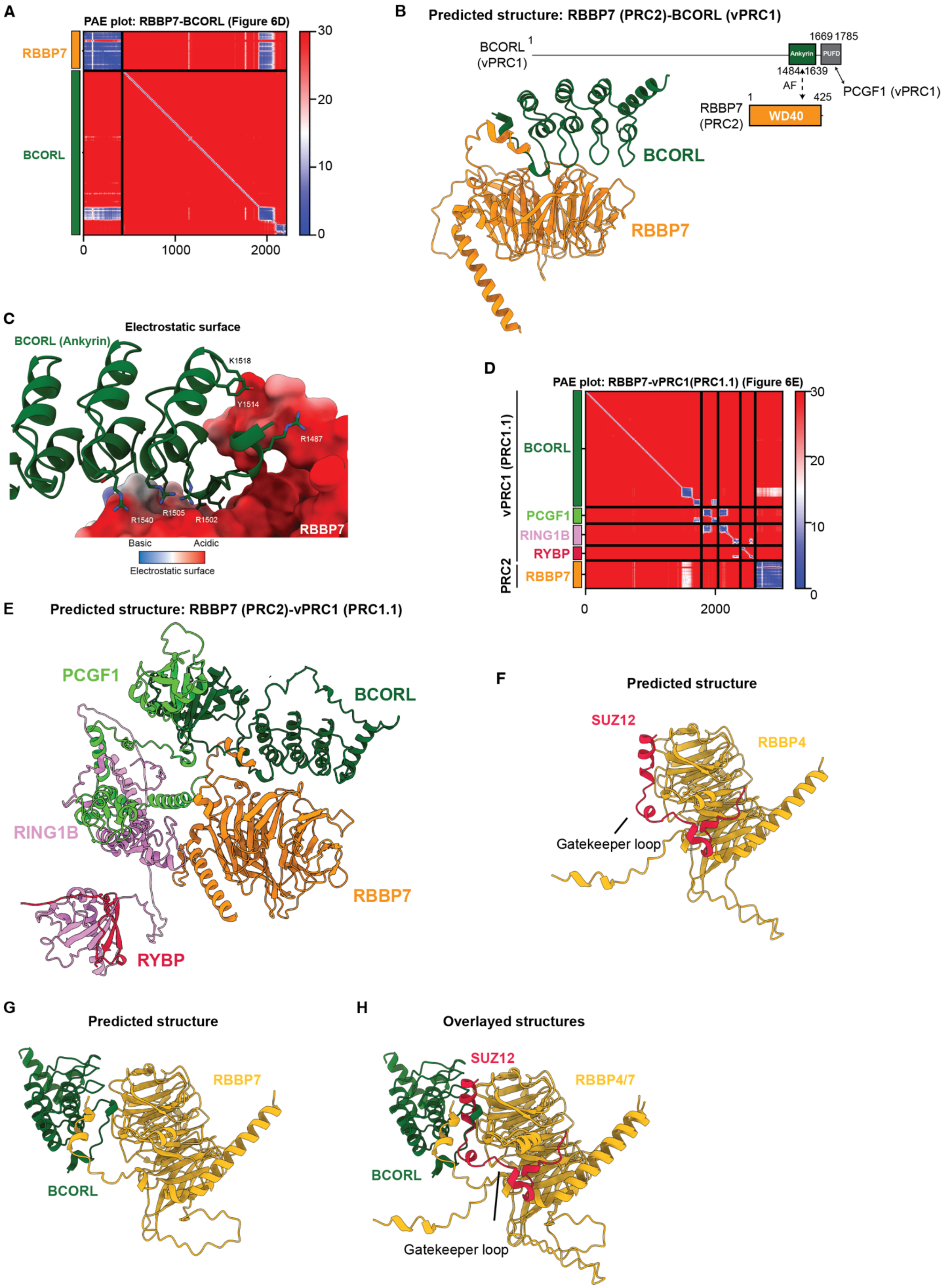
A
PAE plot: RBBP7-BCORL (Figure 6D)
RBBP7
BCORL
0
1000
2000
30
20
10
0
B
Predicted structure: RBBP7 (PRC2)-BCORL (vPRC1)
BCORL
(vPRC1)
1
1669 1785
Ankyrin
PUFD
1484
1639
AF
PCGF1 (vPRC1)
1
425
RBBP7
(PRC2)
WD40
BCORL
RBBP7
C
Electrostatic surface
BCORL (Ankyrin)
K1518
Y1514
R1487
R1540
R1505
R1502
RBBP7
Basic
Acidic
Electrostatic surface
D
PAE plot: RBBP7-vPRC1(PRC1.1) (Figure 6E)
vPRC1 (PRC1.1)
PRC2
BCORL
PCGF1
RING1B
RYBP
RBBP7
0
2000
30
20
10
0
E
Predicted structure: RBBP7 (PRC2)-vPRC1 (PRC1.1)
PCGF1
BCORL
RING1B
RBBP7
RYBP
F
Predicted structure
SUZ12
RBBP4
Gatekeeper loop
G
Predicted structure
RBBP7
BCORL
H
Overlayed structures
SUZ12
RBBP4/7
BCORL
Gatekeeper loop

Fig. S11. AlphaFold multimer predicted interactions between vPRC1 and PRC2 subunits. **A,** The Predicted Aligned Error (PAE) plot of the RBBP7-BCORL interaction. **B,** The predicted structure of the ankyrin repeats (amino acids 1484-1639) of the vPRC1 subunit, BCOR, bound to the RBBP7 subunit of PRC2. Map at the top shows the interacting regions of BCOR with RBBP7 and PCGF1. **C,** Zoomed in view of the BCORL interaction with RBBP7 highlighting the interaction of BCORL lysines with the acidic surface of RBBP9. **D,** The PAE plot of the predicted structure of RBBP7-vPRC1 complex. **E,** AlphaFold predicted structure of the entire vPRC1 complex bound to RBBP7. **F-H,** The predicted interaction interface between RBBP4-SUZ12 (F) and RBBP4-BCORL (G) highlighting the clash between SUZ12 gatekeeper loop and BCORL (H).

Table S1. List of reagents used in this study.

Reagents Used	
DMEM	Invitrogen 11995065
Glutamine	Invitrogen 25030081
Pen Strep	Invitrogen 15140-122
Fetal Bovine Serum	Invitrogen 10437036
Trypsin-EDTA 0.05%	ThermoFisher 25300054
Doxycycline	Sigma, D9891
Opti-MEM	ThermoFisher 31985062
Lipofectamine	ThermoFisher 11668030
Neon Transfection System	ThermoFisher MPK1025
GeneArt™ Precision gRNA Synthesis Kit	ThermoFisher A29377
Cas9 protein	Gift from Jichuan Zhang, (Initiative for Genome Editing and Neurodegeneration core in the Department of Cell Biology at Harvard Medical School)
Puromycin	ThermoFisher A1113803
Hygromycin	ThermoFisher 10687010
RIPA buffer	Sigma R0278
Biorad Protein Assay	Biorad 5000006
4–15% Mini-PROTEAN® TGX™ Precast Protein Gels	BioRad 4561085

Licor IRDye secondary antibody	Licor 925-68071 and 925-32210
ECL IgG, HRP-linked whole Ab	GE Healthcare NA934 and NA931
rNeasy Mini kit	Qiagen 74104,
Random Hexamers	Invitrogen N8080127
SuperScript III	ThermoFisher, 18080044
rNasin® Plus rNase Inhibitor	Promega N2615
GoTaq® G2 Green Master Mix	Promega M7823
HiSpeed Plasmid Maxi Kit	Qiagen 12663
PEG-it Virus precipitation solution	SBI LV810A-1
Polybrene	Sigma H9268
Nunc™ Lab-Tek™ II Chamber Slide™ System	154526PK
Vectashield HardSet Antifade Mounting Medium with DAPI	Vector Labs H-1200-10
Pierce™ 16% Formaldehyde (w/v), Methanol-free	ThermoFisher 28908
QIAQuick PCR Purification Kit	Qiagen 28104
NucleoSpin® Gel and PCR Clean-Up	Takara 740609.250
Spike-in Chromatin	Active Motif 53083
Phenol:Chloroform:Isoamyl Alcohol 25:24:1 Saturated with 10 mM Tris, pH 8.0, 1 mM EDTA.	Sigma P2069
Dynabeads Protein A/G beads	ThermoFisher 10003D and 10001D

Table S2. List of the cell lines generated and the sgRNAs used for this study.

Cell Lines	sgRNAs
**Cell Line 1**: *hUBC-mCherry-2A-rTetR-CBX7; 5xtetO-EF1-H2B-CITRINE-PolyA at WT1 (chr11)*	AATTATGCACCTTCGAGGCC
**Cell Line 1.1A** *hUBC-mCherry-2A-rTetR-CBX7-3xFlag; 5xtetO-EF1-H2B-CITRINE-PolyA at WT1 (chr11)*	AATTATGCACCTTCGAGGCC
**Cell Line 1.1B**. *hUBC-mCherry-2A-rTetR-CBX7Δ; 5xtetO-EF1-H2B-CITRINE-PolyA at WT1 (*chr11)	rTetR: GATGTGAGAGGAGAGCACAG; GCCATGACTCGCCTTCCAGG
**Cell Line 1.2**. *hUBC-mCherry-2A-rTetR-CBX7; 5xtetO-EF1-H2B-CITRINE-PolyA at WT1 (*chr11); *ΔEED*	EED: TTGCCACCAGAGTGTCCGTC
**Cell Line 1.3**. *hUBC-mCherry-2A-rTetR-CBX7; 5xtetO-EF1-H2B-CITRINE-PolyA at WT1 (*chr11); *ΔSUZ12*	SUZ12: CGAAGAGTGAACTGCAACGT
**Cell Line 1.4**. *hUBC-mCherry-2A-rTetR-CBX7; 5xtetO-EF1-H2B-CITRINE-PolyA at WT1 (*chr11); *ΔRING1A and RING1B*	RING1A: GTTCTGAATGCAGTGACCGA RING1B: AATTCACTGTGTAGACTTCG
**Cell Line 1.5**. *hUBC-mCherry-2A-rTetR-CBX7; 5xtetO-EF1-H2B-CITRINE-PolyA at WT1 (*chr11); *ΔMTF2*	MTF2: AGAAGAAGAAGCATTTGTTT
**Cell Line 1.6**. *hUBC-mCherry-2A-rTetR-CBX7; 5xtetO-EF1-H2B-CITRINE-PolyA at WT1 (*chr11); *ΔJARID2*	JARID2: ACAGGTGCTATCCCTCGGGG

**Cell Line 1.7**. *hUBC-mCherry-2A-rTetR-CBX7; 5xtetO-EF1-H2B-CITRINE-PolyA at WT1 (*chr11); *ΔPHF1*	PHF1: TCTTCTTACAGCAAACACTG; GTTTGTTTGGTCTCCATGCT
**Cell Line 1.8.** *hUBC-mCherry-2A-rTetR-CBX7; 5xtetO-EF1-H2B-CITRINE-PolyA at WT1 (*chr11); *ΔPHF19*	PHF19: CCTGGCCCTCTATAATCTGG; GCGTCCCACCCAACCCGCCA
**Cell Line 1.9.** *hUBC-mCherry-2A-rTetR-CBX7; 5xtetO-EF1-H2B-CITRINE-PolyA at WT1 (*chr11); *ΔEZH1 and EZH2*	EZH1: TCGATGTTGGGTGTGCACTG; GCGTTTATATACATTAGGGG EZH2: ACCCCACCAAAACGTCCAGG; TGTCATAGTAAGTGCCAATG
**Cell Line 2***: hUBC-mCherry-2A-rTetR-CBX7; 5xtetO-EF1-H2B-CITRINE-PolyA at EN2 (Chr7)*	AAGCTTCCTGAAACACCTTG
**Cell Line 3**: *hUBC-mCherry-2A-rTetR-CBX7; 5xtetO-EF1-H2B-CITRINE-PolyA at HOXD11 (Chr2)*	TAACGAACAGTCAACACTCG
**Cell Line 4**: *hUBC-mCherry-2A-rTetR-CBX7; 5xtetO-EF1-H2B-CITRINE-PolyA at HOXB4 (Chr17)*	AAGGTCCCTGGTTGACGCTA
**Cell Line 5**: *hUBC-mCherry-2A-rTetR-CBX7; 5xtetO-EF1-H2B-CITRINE-PolyA at TFRC (Chr3)*	AACTGACCTTCAGGCCCGTA
**Cell Line 5.1A**: *hUBC-mCherry-2A-rTetR-CBX7-3xFlag; 5xtetO-EF1-H2B-CITRINE-PolyA at TFRC (Chr3)*	AACTGACCTTCAGGCCCGTA
**Cell Line 6**: *hUBC-mCherry-2A-rTetR-CBX7; 5xtetO-EF1-H2B-CITRINE-PolyA at B2M (Chr15)*	CGTGAAGCCAGCATAGTACT

Table S3. List of the rescue construct cell lines generated

Rescue Cell Lines	Selection Marker/sgRNA used
**Cell Line 1.2.1** *hUBC-mCherry-2A-rTetR-CBX7; 5xtetO-EF1-H2B-CITRINE-PolyA at WT1 (*chr11); *ΔEED; HA-WT-EED*	PUROMYCIN
**Cell Line 1.2.2** *hUBC-mCherry-2A-rTetR-CBX7; 5xtetO-EF1-H2B-CITRINE-PolyA at WT1 (*chr11); *ΔEED; HA-EED-3A*	PUROMYCIN
**Cell Line 1.5.1**. *hUBC-mCherry-2A-rTetR-CBX7; 5xtetO-EF1-H2B-CITRINE-PolyA at WT1 (*chr11); *ΔMTF2; Flag-WT MTF2*	PUROMYCIN
**Cell Line 1.5.2**. *hUBC-mCherry-2A-rTetR-CBX7; 5xtetO-EF1-H2B-CITRINE-PolyA at WT1 (*chr11); *ΔMTF2; Flag- MTF2-EH*	PUROMYCIN
**Cell Line 1.1C** *hUBC-mCherry-2A-rTetR-CBX7; 5xtetO-EF1-H2B-CITRINE-PolyA at WT1 (*chr11); *WT-RYBP-Flag*	HYGROMYCIN
**Cell Line 1.1D** *hUBC-mCherry-2A-rTetR-CBX7; 5xtetO-EF1-H2B-CITRINE-PolyA at WT1 (*chr11); *RYBP-2A-Flag*	HYGROMYCIN
**Cell Line 1.2.2.1** *hUBC-mCherry-2A-rTetR-CBX7; 5xtetO-EF1-H2B-CITRINE-PolyA at WT1 (*chr11); *ΔEED; HA-EED-3A; WT-RYBP-Flag*	HYGROMYCIN
**Cell Line 1.2.2.2** *hUBC-mCherry-2A-rTetR-CBX7; 5xtetO-EF1-H2B-CITRINE-PolyA at WT1 (*chr11); *ΔEED; HA-EED-3A; RYBP-2A-Flag*	HYGROMYCIN

**Cell Line 1.2.2.3** *hUBC-mCherry-2A-rTetR-CBX7;*<br>*5xtetO-EF1-H2B-CITRINE-PolyA at WT1 (*chr11);<br>*ΔEED; HA-EED-3A; ΔEZH1 and EZH2*	EZH1:<br>TCGATGTTGGGTGTGCACTG;<br>GCGTTTATATACATTAGGGG<br>EZH2:<br>ACCCCACCAAAACGTCCAGG;<br>TGTCATAGTAAGTGCCAATG
**Cell Line 1.2.2.4** *hUBC-mCherry-2A-rTetR-CBX7;*<br>*5xtetO-EF1-H2B-CITRINE-PolyA at WT1 (*chr11);<br>*ΔEED; HA-EED-3A; ΔAEBP2*	AEBP2: CTGGTCCCAACAACAATTAT;<br>ATACATGTAGATGGTCAGCG

Table S4. List of Genotyping Primers used in this study.

**<u>Cell Line 1.7</u>.** *hUBC-mCherry-2A-rTetR-CBX7;* *5xtetO-EF1-H2B-CITRINE-PolyA at WT1 (*chr11); *ΔPHF1*	Genotyping Primer
Primer F	CTGGCTCTTAAAATGCCTCTGT
Primer R	TGGACCAGTGACCTGGTGA
**<u>Cell Line 1.8</u>.** *hUBC-mCherry-2A-rTetR-CBX7;* *5xtetO-EF1-H2B-CITRINE-PolyA at WT1 (*chr11); *ΔPHF19*	
Primer F	AGGCTGCCACCTCACCTGGTCC
Primer R	CTGACCCAGGCTTGCTCTTTC

Table S5. List of Antibodies used in this study.

Antibodies	Source	Cat#	Application
Anti-RING1B	Cell Signaling Technology	5694S	WB, 1:200
Anti-RING1A	Cell Signaling Technology	13069S	WB, 1:200
Anti-BETA ACTIN	Abcam	mAbcam8224	WB, 1:1000
Anti-FLAG	Sigma	F3165	WB, 1:5000<br>ChIP, 4ug
Anti-SUZ12	Cell Signaling Technology	3737	IB, 1:1000
Anti-H3K27me3	Cell Signaling Technology	9733	WB, 1:1000<br>ChIP, 3ug
Anti-H2AK119ub1	Cell Signaling Technology	8240T	WB, 1:1000<br>ChIP, 2ug
Anti-MTF2	ProteinTech	16208-1-AP	WB, 1:100<br>ChIP, 4ug
Anti-JARID2	Novus Biologics	NB100-2214SS	WB, 1:500
Anti-HA	ThermoFisher	26183-HRP	WB, 1:2000
Anti-HA Tag Monoclonal Antibody (2-2.2.14)	Thermo-Fisher	26183	ChIP, 2ug
Anti-CBX7	Abcam	ab21873	WB, 1:1000

Anti-Histone H2Av antibody	Active Motif	61686	ChIP-Seq Spike in: 2ug
Anti-B2M	Cell Signaling	12851	WB, 1:200
Anti- TFRC	Cell Signaling	13113T	WB, 1:200
Anti-RYBP	Sigma	PRS2227	WB, 1:200
Anti-SP1	Cell Signaling	5931	WB, 1:200
Anti-PAX2	Cell Signaling	9666	WB, 1:100
Anti-EZH1	Cell Signaling	42088	WB, 1:500
Anti-EZH2	Cell Signaling	5246	WB, 1:500
Anti-AEBP2	Cell Signaling	14129	WB, 1:100

Table S6. qRT-PCR primers used in this study.

Primer Name	Primer Sequence
GAPDH qRT-PCR F	CAATGACCCCTTCATTGACC
GAPDH qRT-PCR R	TTGATTTTGGAGGGATCTCG
TFRC qRT-PCR F	ACCGGCACCATCAAGCT
TFRC qRT-PCR R	TGATCACGCCAGACTTTGC
B2M qRT-PCR F	ACTGAATTCACCCCCACTGA
B2M qRT-PCR R	CCTCCATGATGCTGCTTACA
WT1 qRT-PCR F	TACACACGCACGGTGTCTTCA
WT1 qRT-PCR R	CTCAGATGCCGACCGTACAAG

Table S7. ChIP-qPCR primers used in this study.

Primer Name	Primer Sequence
TetO F	ACGTATGTCGAGGTAGGCGT
TetO R	CTAGGCACCGGTTCAATTGC
CITRINE F	CGACTTCTTCAAGTCCGCCA
CITRINE R	CTTGTAGTTGCCGTCGTCCT
H2B F	CGTGTCCGAGGGTACTAAGG
H2B R	CTTGTGGCCGTTTACGTCG
GAPDH ChIP-qPCR F	AACAGCCTCAAGATCATCAGC
GAPDH ChIP-qPCR R	GGATGATGTTCTGGAGAGCC
PCDH10 ChIP-qPCR F	GGATGGCAACCGATTCGCTGA
PCDH10 ChIP-qPCR R	ACCTCCTCCG TCCACCGCGGT
MYT1 ChIP-qPCR F	ACAAAGGCAGATACCCAACG
MYT1 ChIP-qPCR R	GCAGTTTCAAAAAGCCATCC
SOX6 ChIP-qPCR F	GGGATAGCACGTGAGGATGG
SOX6 ChIP-qPCR R	GGACACCAGACAAGCCTACC
PTF1A ChIP-qPCR F	ATGGACGCGGTGTTGCTGGA
PTF1A ChIP-qPCR R	CGTGAAGACTGGTCGGTGAA
HOXA3 ChIP-qPCR F	GTGCCAATGTGCGCCCTCAC

HOXA3 ChIP-qPCR R	GAGCTGTCGTAGTAGGTCGC
HOXA10 ChIP-qPCR F	CTCTTTCGCGCAGAACATCA
HOXA10 ChIP-qPCR R	TGGCCGAGACTTTGGGGCAT
**WT1**	
WT1 ChIP-qCPR +1.5F	TGCATAAACGTTGTCGCCATT
WT1 ChIP-qCPR +1.5R	AAGTGCGCCCTTCGAGTAAG
WT1 ChIP-qCPR +3F	CTAAGTGCTGCTGACTCCAAT
WT1 ChIP-qCPR +3R	TTTGTGGGTTCCAGAGGTCG
WT1 ChIP-qCPR +4F	CCAGGCCAGGATGTTTCCTAA
WT1 ChIP-qCPR +4R	GTGTCCTAGAGCGGAGAGTC
WT1 ChIP-qCPR +5F	GGGACCGGGATGTTTTTGGA
WT1 ChIP-qCPR +5R	TAAGGTAGGAGCGGCCTGAA
WT1 ChIP-qCPR -1.5F	GGAATTCCAGATGGTGCGCT
WT1 ChIP-qCPR -1.5R	GGCCAGAGCAGATACGTAGG
WT1 ChIP-qCPR -3F	TATAAACAGCTGCCCTGCCG
WT1 ChIP-qCPR -3R	GTCCAGATGCAGGAAGGGTT
WT1 ChIP-qCPR -4F	TGTGGTAACTCCAGGAAGAGGA
WT1 ChIP-qCPR -4R	AGCGTATGTCAAGGACATTGGT
WT1 ChIP-qCPR -5F	CAGCGTTTGGATTCGGGTTC
WT1 ChIP-qCPR -5R	CGCCCGACCCCGTAATTTT
**TFRC**	

TFRC ChIP-qPCR +0.5F	TGGGTTGCTTTCTTGCATTGT
TFRC ChIP-qPCR +0.5R	CAATCACACCCTCTCCCTCC
TFRC ChIP-qPCR +1F	CGTACGTGCCTCAGGAAGTG
TFRC ChIP-qPCR +1R	GTTCTAGAAGCCCGCACTCA
TFRC ChIP-qPCR +2F	GGACAAAGCTGTCCCCGATT
TFRC ChIP-qPCR +2R	AGAATCCACACACAAGGCGA
TFRC ChIP-qPCR +3F	CATGGTTCAAAACGTGGGGC
TFRC ChIP-qPCR +3R	TGGTGTTCTCAATGGTGACTGAA
TFRC ChIP-qPCR -0.5F	TAAGGTAGGCCCTCTGTGGAT
TFRC ChIP-qPCR -0.5R	CAGGAGCATTGCTGCACCTTTA
TFRC ChIP-qPCR -1F	AAACCTATGTCCTCCATGAGGCT
TFRC ChIP-qPCR -1R	TGCCTTTCCTTGACTGAAGTATC
TFRC ChIP-qPCR -2F	CCAGGCTCAGGAAAGTTGAGA
TFRC ChIP-qPCR -2R	AAGATACCATGAGCTGTGGGG
TFRC ChIP-qPCR -3F	CCTGCACTGTTGTTTCCAGC
TFRC ChIP-qPCR -3R	GGAGCCTGTGGTGTGTGTTA
**B2M**	
B2M ChIP-qPCR +0.5F	CTATGGTAACCACCGCCTGG

B2M ChIP-qPCR +0.5R	AGGGACATAAGCTTGGCTGG
B2M ChIP-qPCR +1F	GAGGCCACTTGGTATTCTAACC
B2M ChIP-qPCR +1R	GTGTGACCCAGCACATTACA
B2M ChIP-qPCR +2F	CACCTCCCCTAGCTATGTCCTTT
B2M ChIP-qPCR +2R	CATGAGGAAACTTTAGGGTTGATGG
B2M ChIP-qPCR +3F	GGACTCCACCACCACGAAAT
B2M ChIP-qPCR +3R	AAGACAAAGGGCTCGGCAAT
B2M ChIP-qPCR +4F	GAGATGTCTCGCTCCGTGG
B2M ChIP-qPCR +4R	AGACTCACGCTGGATAGCCT
B2M ChIP-qPCR -0.5F	CAGTCTGGGCTGTTTGTATCT
B2M ChIP-qPCR -0.5R	GCTTGGTGTGCCCTCTAAT
B2M ChIP-qPCR -1F	TCAAGCTCACTAATTCTTTCTTCCA
B2M ChIP-qPCR -1R	ATACTGACATACATAAGGGTGCAT
B2M ChIP-qPCR -2F	TCCACTGGAAAGTCTGCTGC
B2M ChIP-qPCR -2R	ACAAACTCCCAAGGTCAAGAA
B2M ChIP-qPCR -3F	GTGCCACTATGTCTGGCTAAT
B2M ChIP-qPCR -3R	GGTGAAAGTGCTGTCTCTACAA
B2M ChIP-qPCR -4F	AGTCCCAGCTACTCAGGAGG
B2M ChIP-qPCR -4R	TCTGCACTATAGATCAAATGGCTC

Table S8. List of data sources from public databases.

File Name	GEO Accession #/Source Information
hESCs H3K27me3 ChIP-Seq	GSM1185386
hESCs RNA-seq	GSM672836
HEK293FT H3K27me3 ChIP-Seq	GSM4239945
HEK293FT H2AK119ub1 ChIP-Seq	GSM4502558
HEK293FT RNA-seq	GSM5343713
cMYC ChIP	GSM3360524
HEK293 Bisulfite Seq	GSM683769
CGIs	UCSC Genome browser
Human Protein Atlas	proteinatlas.org
Transcription Factor ChIP	UCSC Genome browser
MTF2 PDB file	10.2210/pdb5XFR/pdb Li et al. (47)
EED PDB file	10.2210/pdb3IIW/pdb Margueron et al. (28)

Table S9. List of Softwares used in this study

bowtie2 (v2.2.9)
deeptool (v3.0.2)
Samtool (v1.3.1)
Integrative Genomics Viewer (v2.12.0)
BD Biosciences-FACsDIVA
BD Biosciences CellQuestPro
FlowJo (v10.5.3)
NIS-Elements imaging software
ImageJ (v1.0)
GraphPad Prism 7.0c
https://benchling.com
https://chopchop.cbu.uib.no/
UCSF Chimera X-1.6.1
localColabFold (AlphaFold2-Multimer v3)

Table S10. Summary of IP-MS results.

Mass spectrometry identification of proteins enriched in rTetR-CBX7-3xFlag immunoprecipitations.

Table S11. List of AlphaFold Multimer predictions.

Interactions are ranked based on quality control scores. See Methods for definitions of ranking criteria.
